# Supplementary material for: Designing and Developing a Population/Literature-Based Westernized Diet Index (WDI) and Its Relevance for Cardiometabolic Health
Source: Nutrients. 2025 Jul 14;17(14):2314. doi: 10.3390/nu17142314 (PMC12300042; doi:10.3390/nu17142314)
Supplement: Supplementary file 1 [file nutrients-17-02314-s001.zip › nutrients-3678207-supplementary.pdf]

WDI Supplementary tables:

Supplementary Table S1. Expanded version of the food groups/items used to develop the WDI.

#	Food group*	Detailed components
**1**	**Calorie**, **Energy**	Calorie as a component
**2**	**Fiber**	Total fibers, Soluble fibers, insoluble fibers
**3**	**Whole grains**, **Potato**	Brown rice, Oats, Barley, Bulgur, Farro, Millet, Whole wheat, Whole rye, Potatoes (including sweet potatoes and yams)
**4**	**Carbohydrates**	Carbohydrate as a component
**5**	**Refined grains**	White rice, White bread, White pasta, White flour, Cornflakes, Rice cakes, Crackers, Pita bread, Bagels, Croissants, Pretzels, Couscous, Naan
**6**	**Legumes**	Legumes, Lentils, Chickpeas, Black beans, Kidney beans, Pinto beans, Navy beans, Cannellini beans, Adzuki beans, Mung beans, Lima beans, Butter beans, Fava beans, Soybeans, Green peas, Split peas, Black-eyed peas, Pigeon peas, Edamame, Lupins, Broad beans
**7**	**Nuts and seed**	Nuts, Almonds, Walnuts, Cashews, Pecans, Pistachios, Hazelnuts, Macadamia nuts, Brazil nuts, Pine nuts, Peanuts (technically a legume), Chestnuts, Seeds, Chia seeds, Flax seeds, Pumpkin seeds, Sunflower seeds, Sesame seeds, Hemp seeds, Poppy seeds, Mustard seeds, Caraway seeds, Cumin seeds, Fennel seeds, Coriander seeds, Celery seeds, Nigella seeds, Fenugreek seeds, Basil seeds, Safflower seeds, Watermelon seeds, Grape seeds, Quinoa
**8**	**Oils (olive**, **Rapeseed**, **Unsaturated nut oils)**	Olive oil, Olive, Canola oil (rapeseed oil), Avocado oil, Walnut oil, Almond oil, Macadamia nut oil, Hazelnut oil, Peanut oil, Sesame oil, Sunflower oil, Safflower oil, Grapeseed oil, Flaxseed oil, Pumpkin seed oil, Hemp seed oil, Chia seed oil, Rice bran oil, Soybean oil, Corn oil, Cottonseed oil
**9**	**Refined fats and oils**, **Margarine**, **Hydrogenated fats**	Margarine, Vegetable shortening, Partially hydrogenated oils, Palm oil, Palm kernel oil, Cocoa butter, Cottonseed oil, Soybean oil (hydrogenated), Canola oil (hydrogenated), Peanut oil (hydrogenated), Coconut oil (refined), Butter (processed), Clarified butter (ghee), Salad dressings (with hydrogenated oils)
**10**	**Soft drinks (nectar**, **Soda)**	Peach nectar, Apricot nectar, Mango nectar, Guava nectar, Pear nectar, Passion fruit nectar, Cherry nectar, Pineapple nectar, Strawberry nectar, Papaya 5nectar, Grape nectar, Apple nectar, Plum nectar, Kiwi nectar, Lychee nectar, Orange soda, Cola, Lemon-lime soda, Root beer, Ginger ale, Cream soda, Grape soda, Orange cream soda, Cherry soda, Birch beer, Dr. Pepper, Mountain Dew, Sprite, Fanta, Pepsi, Coca-Cola, Sierra Mist, Sunkist, 7-Up, Fresca, Blue raspberry soda, Fruit punch soda, Vanilla cream soda
**11**	**Sodium**	Table salt, Soy sauce, Teriyaki sauce, Barbecue sauce, Ketchup, Canned soups, Canned vegetables, Pickles, Salted popcorn
**12**	**Coffee**, **Tea**, **waters**	Coffee (various types: black, espresso, latte, etc.), Black tea, Green tea, White tea, Oolong tea, Herbal tea (various flavors: chamomile, peppermint, etc.), Rooibos tea, Matcha tea, Chai tea, Iced coffee, Iced tea (various flavors: lemon, peach, etc.), Cold brew coffee, Filtered water, Mineral water, Spring water, Flavored water (various flavors: lemon, cucumber, berry, etc.), Sparkling water
**13**	**Protein**	Protein as a component
**14**	**Diet drinks (zero calorie beverages)**	Diet soda (various flavors), Diet cola, Diet lemon-lime soda, Diet root beer, Diet ginger ale, Diet cream soda, Diet grape soda, Diet orange soda, Diet cherry soda, Diet birch beer, Diet Dr. Pepper, Diet Mountain Dew,

		Diet Sprite, Diet Fanta, Diet Pepsi, Diet Coca-Cola, Diet Sierra Mist, Diet Sunkist, Diet 7-Up, Diet Fresca, Diet Blue raspberry soda, Diet fruit punch soda
**15**	**Alcohol and wine**	Alcoholic drinks and wine
**16**	**Supplements**	Any kind of supplements
**17**	**Vitamins and minerals**	Vitamins and minerals from diet
**18**	**Secondary plant metabolites**	Turmeric, Curcumin, Ginger, Garlic, Ginseng, Resveratrol, Quercetin, Lycopene, Anthocyanins, Flavonoids, Catechins, Glucosinolates, Polyphenols, Saponins, Tannins, Phytosterols, Capsaicin, Chlorophyll, Allicin, Lutein, Zeaxanthin, Isoflavones, Ellagic acid, Apigenin, Beta-glucan, Caffeic acid, Ferulic acid, Kaempferol, Lignans, Myricetin, Pterostilbene, Rosmarinic acid, Sulforaphane
**19**	**Total fat**	Total fat or fat as a component
**20**	**Processed foods**, **Fast foods**, **NOVA classification**	Chicken nuggets, Microwave meals, Instant noodles, Canned pasta, Frozen pizzas, Potato chips, Cheese puffs, Pastries, Sugary cereals, Sweetened yogurt, Breakfast bars, Snack cakes
**21**	**Cholesterol**, **Saturated Fatty Acids (SFA)**, **Trans fat**	Cholesterol, SFA, Trans fat, Lard, Beef tallow, Cream, Coconut oil, Pork belly, Chicken skin, Duck fat
**22**	**MUFA, PUFA**	Monounsaturated Fatty Acids (MUFA), Polyunsaturated Fatty Acids (PUFA)
**23**	**Simple sugars**, **Sugar beverages**	Table sugar, Honey, Maple syrup, Corn syrup, Sweetened teas, Sweetened coffee drinks, Flavored milks, Sports drinks, Candy, Jelly, Jam, Ice cream, Cake, Cookies, Doughnuts, Pies, Chocolate, Biscuits, Candy bars
**24**	**Fruits**	Fruits as a component
**25**	**Vegetables**	Vegetable as a component
**26**	**Processed meat**	Processed red meat, Processed meat, Bacon, Ham, Salami, Pepperoni, Sausages, Hot dogs, Corned beef, Pastrami, Bologna, Chorizo, Mortadella, Prosciutto, Kielbasa, Soppresata, Bratwurst, Blood sausage, Beef jerky, Pâté, Meatballs, Meatloaf
**27**	**Red meat**	Beef steak, Ground beef, Lamb chops, Pork chops, Venison, Buffalo, Elk, Goat, Mutton, Beef ribs, Pork ribs, Veal, Brisket, Roast beef, Prime rib, Filet mignon, T-bone steak, Porterhouse steak, Ribeye steak, Sirloin steak
**28**	**White meat**	Chicken breast, Chicken thighs, Chicken wings, Chicken drumsticks, Turkey breast, Turkey thighs, Turkey wings, Ground turkey, Duck breast, Goose, Quail, Pheasant, Rabbit, Ostrich
**29**	**Fish**	Fatty fish, lean fish, Salmon, Tuna, Cod, Haddock, Halibut, Mackerel, Sardines, Anchovies, Trout, Bass, Flounder, Sole, Snapper, Grouper, Herring, Swordfish, Tilapia, Catfish, Perch, Pollock
**30**	**Dairy**	Milk, Cheese, Yogurt, Cream, Cottage cheese, Sour cream, Cream cheese, Buttermilk, Kefir, Ghee, Skyr, Fromage blanc, Ricotta, Paneer, Whey protein, Casein protein, Lactose-free milk

* global mean ± SDs or population based mean ± SDs are needed to calculate the final score using the index

Supplementary Table S2. Latest versions of MetS definitions, cut-off points, and components presented by different health organizations

	WHO, 1998 [71]	EGIR, 1999 [72]	NCEP: ATPIII, 2002 [10, 73]	AACE, 2003 [74]	IDF, 2006 [75]
**Mandatory component/s**	GI, IGT or DM, and/or IR together with two or more of the following components:	Top 25% of the fasting insulin values in non-diabetics with two of the following components:	Three or more of the following components:	IGT and two or more of the following components:	Central obesity as defined by ethnic/racial, specific WC, and two of the following components:
**Blood pressure**	≥ 140/90 mm Hg	≥140/90 mm Hg Or medications to control HT	≥130/85 mm Hg	≥130/85 mm Hg	≥130/85 mm Hg
**Triglycerides**	≥150 mg dl^{-1} And/or Low HDL-c: Males <35 mg dl^{-1}; Female < 39 mg dl^{-1}	≥177 mg dl^{-1}	≥150 mg dl^{-1}	≥150 mg dl^{-1}	≥150 mg dl^{-1}
**Central obesity**	Males: WHR >90 cm; Females: WHR >85 cm, And/or BMI >30 kg m^{-2}	Males: WC ≥94 cm; Females: ≥80 cm	Males WC > 101 cm; Females: WC > 88.9 cm	-	-
**Fasting blood glucose**	-	≥110 mg dl^{-1}	≥110 mg dl^{-1}		FBG ≥100 mg dl^{-1}
**High-density lipoprotein cholesterol**	-	< 39 mg dl^{-1}	Males <40 mg dl^{-1}; Female<50 mg dl^{-1}	Males <40 mg dl^{-1}; Females <50 mg dl^{-1}	Males <40 mg dl^{-1}; Females <50 mg dl^{-1}

GI: glucose intolerance; IGT: Impaired glucose tolerance; DM: diabetes mellitus; IR: insulin resistance; WHR: waist to hip ratio; WC: waist circumference; HT: hypertension

Supplementary Table S3. Summary of search strategy. Taken steps to design a search strategy to capture relevant studies in the literature aligned with WDI objectives.

#	**Search terms**
**#1**	"Metabolic Syndrome"[Mesh]
**#2**	"Hexoses"[MeSH Terms] OR "Oils"[MeSH] OR "Cholesterol, Dietary"[MeSH Terms] OR "Dietary Sugars"[MeSH Terms] OR "Maltose"[MeSH Terms] OR "Sucrose"[MeSH Terms] OR "Fructose"[MeSH Terms] OR "Dietary fats"[MeSH Terms] OR "Sodium, Dietary"[Mesh] OR "Food, Processed"[Mesh] OR Meat[Mesh] OR "Dietary Fiber"[Mesh] OR "Diet, Western"[Mesh] OR Fruit[Mesh] OR Vegetables[Mesh] OR "Food Additives"[Mesh] OR Candy[Mesh] OR Antioxidants[Mesh] OR "Fast Foods"[Mesh] OR Beverages[Mesh] OR "Dietary Supplements"[Mesh] OR "Emulsifying Agents"[Mesh] OR Snacks[Mesh] OR "Potassium, Dietary"[Mesh] OR "Vitamin A"[Mesh]
**#3**	"Blood Glucose"[MeSH Terms]
**#4**	#2 NOT #3
**#5**	#1 AND #4
**#6**	("case reports"[Publication Type] OR "review"[Publication Type] OR "systematic review"[Publication Type] OR "Meta-Analysis"[Publication Type] OR "Comment"[Publication Type] OR "Congress"[Publication Type] OR "Editorial"[Publication Type] OR "books"[MeSH Terms] OR "retracted publication"[Publication Type])
**#7**	#5 NOT #6
**#8**	(excludepreprints[Filter]) AND (humans[Filter]) AND (english[Filter])
**#9**	#7 AND #8
**Hits**	1581

Supplementary Table S4. Articles used for the present investigation.

comments	author	publication year	title
1	Zujko, M. E.; Rożniata, M.; Zujko, K.	2021	Individual Diet Modification Reduces the Metabolic Syndrome in Patients Before Pharmacological Treatment
2	Aboonabi, A.; Aboonabi, A.	2020	Anthocyanins reduce inflammation and improve glucose and lipid metabolism associated with inhibiting nuclear factor-kappaB activation and increasing PPAR-γ gene expression in metabolic syndrome subjects
3	Acharjee, S.; Zhou, J. R.; Elajami, T. K.; Welty, F. K.	2015	Effect of soy nuts and equol status on blood pressure, lipids and inflammation in postmenopausal women stratified by metabolic syndrome status
4	Ahn, J.; Kim, N. S.; Lee, B. K.; Park, S.	2017	Carbohydrate Intake Exhibited a Positive Association with the Risk of Metabolic Syndrome in Both Semi-Quantitative Food Frequency Questionnaires and 24-Hour Recall in Women
5	Ahola, A. J.; Forsblom, C. M.; Harjutsalo, V.; Groop, P. H.	2021	Nut Consumption Is Associated with Lower Risk of Metabolic Syndrome and Its Components in Type 1 Diabetes
6	Ahola, A. J.; Harjutsalo, V.; Thorn, L. M.; Freese, R.; Forsblom, C.; Mäkimattila, S.; Groop, P. H.	2017	The association between macronutrient intake and the metabolic syndrome and its components in type 1 diabetes
7	Aiello, A.; Di Bona, D.; Candore, G.; Carru, C.; Zinellu, A.; Di Miceli, G.; Nicosia, A.; Gambino, C. M.; Ruisi, P.; Caruso, C.; Vasto, S.; Accardi, G.	2018	Targeting Aging with Functional Food: Pasta with Opuntia Single-Arm Pilot Study
8	Al-Daghri, N. M.; Amer, O. E.; Khattak, M. N. K.; Sabico, S.; Ghouse Ahmed Ansari, M.; Al-Saleh, Y.; Aljohani, N.; Alfawaz, H.; Alokail, M. S.	2019	Effects of different vitamin D supplementation strategies in reversing metabolic syndrome and its component risk factors in adolescents
9	Al-Solaiman, Y.; Jesri, A.; Mountford, W. K.; Lackland, D. T.; Zhao, Y.; Egan, B. M.	2010	DASH lowers blood pressure in obese hypertensives beyond potassium, magnesium and fibre
10	Alves, N. E.; Enes, B. N.; Martino, H. S.; Alfenas Rde, C.; Ribeiro, S. M.	2014	Meal replacement based on Human Ration modulates metabolic risk factors during body weight loss: a randomized controlled trial

11	An, H. J.; Kim, Y.; Seo, Y. G.	2023	Relationship between Coffee, Tea, and Carbonated Beverages and Cardiovascular Risk Factors
12	Angelico, F.; Loffredo, L.; Pignatelli, P.; Augelletti, T.; Carnevale, R.; Pacella, A.; Albanese, F.; Mancini, I.; Di Santo, S.; Del Ben, M.; Violi, F.	2012	Weight loss is associated with improved endothelial dysfunction via NOX2-generated oxidative stress down-regulation in patients with the metabolic syndrome
13	Angelopoulos, T. J.; Lowndes, J.; Sinnett, S.; Rippe, J. M.	2016	Fructose Containing Sugars at Normal Levels of Consumption Do Not Effect Adversely Components of the Metabolic Syndrome and Risk Factors for Cardiovascular Disease
14	Appelhans, B. M.; Baylin, A.; Huang, M. H.; Li, H.; Janssen, I.; Kazlauskaite, R.; Avery, E. F.; Kravitz, H. M.	2017	Beverage Intake and Metabolic Syndrome Risk Over 14 Years: The Study of Women's Health Across the Nation
15	Aquino, S. L. S.; Cunha, A. T. O.; Sena-Evangelista, K. C. M.; Lima, J. G.; Pedrosa, L. F. C.	2023	Vitamin D(3) supplementation had no benefits in patients with metabolic syndrome and vitamin D deficiency: A pilot study
16	Arentoft, J. L.; Hoppe, C.; Andersen, E. W.; Overvad, K.; Tetens, I.	2018	Associations between adherence to the Danish Food-Based Dietary Guidelines and cardiometabolic risk factors in a Danish adult population: the DIPI study
17	Arsic, A.; Takic, M.; Kojadinovic, M.; Petrovic, S.; Paunovic, M.; Vucic, V.; Ristic Medic, D.	2021	Metabolically healthy obesity: is there a link with polyunsaturated fatty acid intake and status?
18	Asadi, Z.; Shafiee, M.; Sadabadi, F.; Saberi-Karimian, M.; Darroudi, S.; Tayefi, M.; Ghazizadeh, H.; Heidari Bakavoli, A.; Moohebati, M.; Esmaeily, H.; Ferns, G. A.; Ghayour-Mobarhan, M.	2019	Association Between dietary patterns and the risk of metabolic syndrome among Iranian population: A cross-sectional study
19	Asgary, S.; Soltani, R.; Zolghadr, M.; Keshvari, M.; Sarrafzadegan, N.	2016	Evaluation of the effects of roselle (Hibiscus sabdariffa L.) on oxidative stress and serum levels of lipids, insulin and hs-CRP in adult patients with metabolic syndrome: a double-blind placebo-controlled clinical trial

20	Asghari, G.; Yuzbashian, E.; Mirmiran, P.; Bahadoran, Z.; Azizi, F.	2016	Prediction of metabolic syndrome by a high intake of energy-dense nutrient-poor snacks in Iranian children and adolescents
21	Asghari, G.; Yuzbashian, E.; Mirmiran, P.; Mahmoodi, B.; Azizi, F.	2015	Fast Food Intake Increases the Incidence of Metabolic Syndrome in Children and Adolescents: Tehran Lipid and Glucose Study
22	Askarpour, M.; Ramezan, M.; Jafari, F.; Nouri, M.; Fattahi, M. R.; Safarpour, A. R.; Shojaei Zarghani, S.; Ghalandari, H.; Abdolahi, N.; Akbarzadeh, M.; Hejazi, N.	2023	The association between low carbohydrate diet and odds of metabolic syndrome in adults: A cross-sectional study
23	Assi, M. J.; Poursalehi, D.; Tirani, S. A.; Shahdadian, F.; Hajhashemy, Z.; Mokhtari, E.; Mohammadi, S.; Saneei, P.	2023	Legumes and nuts intake in relation to metabolic health status, serum brain derived neurotrophic factor and adropin levels in adults
24	Athyros, V. G.; Liberopoulos, E. N.; Mikhailidis, D. P.; Papageorgiou, A. A.; Ganotakis, E. S.; Tziomalos, K.; Kakafika, A. I.; Karagiannis, A.; Lambropoulos, S.; Elisaf, M.	2007	Association of drinking pattern and alcohol beverage type with the prevalence of metabolic syndrome, diabetes, coronary heart disease, stroke, and peripheral arterial disease in a Mediterranean cohort
25	Azadbakht, L.; Esmaillzadeh, A.	2009	Red meat intake is associated with metabolic syndrome and the plasma C-reactive protein concentration in women
26	Azadbakht, L.; Kimiagar, M.; Mehrabi, Y.; Esmaillzadeh, A.; Padyab, M.; Hu, F. B.; Willett, W. C.	2007	Soy inclusion in the diet improves features of the metabolic syndrome: a randomized crossover study in postmenopausal women
27	Babio, N.; Becerra-Tomás, N.; Martínez-González, MÁ; Corella, D.; Estruch, R.; Ros, E.; Sayón-Orea, C.; Fitó, M.; Serra-Majem, L.; Arós, F.; Lamuela-Raventós, R. M.; Lapetra, J.; Gómez-Gracia, E.; Fiol, M.;	2015	Consumption of Yogurt, Low-Fat Milk, and Other Low-Fat Dairy Products Is Associated with Lower Risk of Metabolic Syndrome Incidence in an Elderly Mediterranean Population

	Díaz-López, A.; Sorlí, J. V.; Martínez, J. A.; Salas-Salvadó, J.		
28	Baek, S. Y.; Kim, H. K.	2023	Association of Dietary Sodium-to-Potassium Ratio with Cardiometabolic Risk Factors in Korean Adults: Findings from the Korean National Health and Nutrition Examination Survey
29	Bahadoran, Z.; Mirmiran, P.; Hosseini-Esfahani, F.; Azizi, F.	2013	Fast food consumption and the risk of metabolic syndrome after 3-years of follow-up: Tehran Lipid and Glucose Study
30	Baik, I.; Abbott, R. D.; Curb, J. D.; Shin, C.	2010	Intake of fish and n-3 fatty acids and future risk of metabolic syndrome
31	Baik, I.; Shin, C.	2008	Prospective study of alcohol consumption and metabolic syndrome
32	Bajerska, J.; Mildner-Szkudlarz, S.; Walkowiak, J.	2015	Effects of rye bread enriched with green tea extract on weight maintenance and the characteristics of metabolic syndrome following weight loss: a pilot study
33	Bakhtiary, A.; Yassin, Z.; Hanachi, P.; Rahmat, A.; Ahmad, Z.; Jalali, F.	2012	Effects of soy on metabolic biomarkers of cardiovascular disease in elderly women with metabolic syndrome
34	Balasubramanian, G. V.; Chuah, K. A.; Khor, B. H.; Sualeheen, A.; Yeak, Z. W.; Chinna, K.; Sundram, K.; Karupaiah, T.	2020	Associations of Eating Mode Defined by Dietary Patterns with Cardiometabolic Risk Factors in the Malaysia Lipid Study Population
35	Baldeón, M. E.; Felix, C.; Fornasini, M.; Zertuche, F.; Largo, C.; Paucar, M. J.; Ponce, L.; Rangarajan, S.; Yusuf, S.; López-Jaramillo, P.	2021	Prevalence of metabolic syndrome and diabetes mellitus type-2 and their association with intake of dairy and legume in Andean communities of Ecuador
36	Balk, L.; Hoekstra, T.; Twisk, J.	2009	Relationship between long-term coffee consumption and components of the metabolic syndrome: the Amsterdam Growth and Health Longitudinal Study
37	Barbosa, L. B.; Vasconcelos, N. B. R.; Dos Santos, E. A.; Dos Santos, T. R.; Ataide-Silva, T.; Ferreira, H. D. S.	2023	Ultra-processed food consumption and metabolic syndrome: a cross-sectional study in Quilombola communities of Alagoas, Brazil
38	Barrea, L.; Muscogiuri, G.; Di Somma, C.; Annunziata, G.; Megna,	2018	Coffee consumption, metabolic syndrome and clinical severity of psoriasis: good or bad stuff?

	M.; Falco, A.; Balato, A.; Colao, A.; Savastano, S.		
39	Barrio-Lopez, M. T.; Bes-Rastrollo, M.; Sayon-Orea, C.; Garcia-Lopez, M.; Fernandez-Montero, A.; Gea, A.; Martinez-Gonzalez, M. A.	2013	Different types of alcoholic beverages and incidence of metabolic syndrome and its components in a Mediterranean cohort
40	Barrio-Lopez, M. T.; Martinez-Gonzalez, M. A.; Fernandez-Montero, A.; Beunza, J. J.; Zazpe, I.; Bes-Rastrollo, M.	2013	Prospective study of changes in sugar-sweetened beverage consumption and the incidence of the metabolic syndrome and its components: the SUN cohort
41	Basu, A.; Du, M.; Leyva, M. J.; Sanchez, K.; Betts, N. M.; Wu, M.; Aston, C. E.; Lyons, T. J.	2010	Blueberries decrease cardiovascular risk factors in obese men and women with metabolic syndrome
42	Basu, A.; Fu, D. X.; Wilkinson, M.; Simmons, B.; Wu, M.; Betts, N. M.; Du, M.; Lyons, T. J.	2010	Strawberries decrease atherosclerotic markers in subjects with metabolic syndrome
43	Basu, A.; Wilkinson, M.; Penugonda, K.; Simmons, B.; Betts, N. M.; Lyons, T. J.	2009	Freeze-dried strawberry powder improves lipid profile and lipid peroxidation in women with metabolic syndrome: baseline and post intervention effects
44	Batista, C. C.; Nascimento, L. M.; Lustosa, Lcrs; Rodrigues, B. G. M.; Campelo, V.; Frota, K. M. G.	2021	Metabolic syndrome in adolescents and antioxidant nutrient intake: a cross-sectional study
45	Batista-Jorge, G. C.; Barcala-Jorge, A. S.; Silveira, M. F.; Lelis, D. F.; Andrade, J. M. O.; de Paula, A. M. B.; Guimarães, A. L. S.; Santos, S. H. S.	2020	Oral resveratrol supplementation improves Metabolic Syndrome features in obese patients submitted to a lifestyle-changing program
46	Baxheinrich, A.; Stratmann, B.; Lee-Barkey, Y. H.; Tschoepe, D.; Wahrburg, U.	2012	Effects of a rapeseed oil-enriched hypoenergetic diet with a high content of α-linolenic acid on body weight and cardiovascular risk profile in patients with the metabolic syndrome
47	Becerra-Tomás, N.; Babio, N.; Martínez-González, MÁ; Corella, D.; Estruch, R.; Ros, E.;	2016	Replacing red meat and processed red meat for white meat, fish, legumes or eggs is associated with lower risk of incidence of metabolic syndrome

	Fitó, M.; Serra-Majem, L.; Salaverria, I.; Lamuela-Raventós, R. M.; Lapetra, J.; Gómez-Gracia, E.; Fiol, M.; Toledo, E.; Sorlí, J. V.; Pedret-Llaberia, M. R.; Salas-Salvadó, J.		
48	Bekkouche, L.; Bouchenak, M.; Malaisse, W. J.; Yahia, D. A.	2014	The Mediterranean diet adoption improves metabolic, oxidative, and inflammatory abnormalities in Algerian metabolic syndrome patients
49	Bellien, J.; Bozec, E.; Bounoure, F.; Khettab, H.; Malloizel-Delaunay, J.; Skiba, M.; Iacob, M.; Donnadieu, N.; Coquard, A.; Morio, B.; Laillet, B.; Rigaudière, J. P.; Chardigny, J. M.; Monteil, C.; Vendeville, C.; Mercier, A.; Cailleux, A. F.; Blanchard, A.; Amar, J.; Fezeu, L. K.; Pannier, B.; Bura-Rivière, A.; Boutouyrie, P.; Joannidès, R.	2022	The effect of camelina oil on vascular function in essential hypertensive patients with metabolic syndrome: a randomized, placebo-controlled, double-blind study
50	Bellikci-Koyu, E.; Sarer-Yurekli, B. P.; Karagozlu, C.; Aydin-Kose, F.; Ozgen, A. G.; Buyuktuncer, Z.	2022	Probiotic kefir consumption improves serum apolipoprotein A1 levels in metabolic syndrome patients: a randomized controlled clinical trial
51	Bernini, L. J.; Simão, A. N.; Alfieri, D. F.; Lozovoy, M. A.; Mari, N. L.; de Souza, C. H.; Dichi, I.; Costa, G. N.	2016	Beneficial effects of Bifidobacterium lactis on lipid profile and cytokines in patients with metabolic syndrome: A randomized trial. Effects of probiotics on metabolic syndrome
52	Beydoun, M. A.; Canas, J. A.; Beydoun, H. A.; Chen, X.; Shroff, M. R.; Zonderman, A. B.	2012	Serum antioxidant concentrations and metabolic syndrome are associated among U.S. adolescents in recent national surveys
53	Beydoun, M. A.; Gary, T. L.; Caballero, B. H.; Lawrence, R. S.; Cheskin, L. J.; Wang, Y.	2008	Ethnic differences in dairy and related nutrient consumption among US adults and their association with obesity, central obesity, and the metabolic syndrome
54	Beydoun, M. A.; Shroff, M. R.; Chen, X.; Beydoun, H. A.; Wang, Y.; Zonderman, A. B.	2011	Serum antioxidant status is associated with metabolic syndrome among U.S. adults in recent national surveys

55	Bian, S.; Gao, Y.; Zhang, M.; Wang, X.; Liu, W.; Zhang, D.; Huang, G.	2013	Dietary nutrient intake and metabolic syndrome risk in Chinese adults: a case-control study
56	Blesso, C. N.; Andersen, C. J.; Barona, J.; Volk, B.; Volek, J. S.; Fernandez, M. L.	2013	Effects of carbohydrate restriction and dietary cholesterol provided by eggs on clinical risk factors in metabolic syndrome
57	Bo, S.; Durazzo, M.; Guidi, S.; Carello, M.; Sacerdote, C.; Silli, B.; Rosato, R.; Cassader, M.; Gentile, L.; Pagano, G.	2006	Dietary magnesium and fiber intakes and inflammatory and metabolic indicators in middle-aged subjects from a population-based cohort
58	Bondia-Pons, I.; Pöhö, P.; Bozzetto, L.; Vetrani, C.; Patti, L.; Aura, A. M.; Annuzzi, G.; Hyötyläinen, T.; Rivellese, A. A.; Orešič, M.	2014	Isoenergetic diets differing in their n-3 fatty acid and polyphenol content reflect different plasma and HDL-fraction lipidomic profiles in subjects at high cardiovascular risk
59	Brader, L.; Uusitupa, M.; Dragsted, L. O.; Hermansen, K.	2014	Effects of an isocaloric healthy Nordic diet on ambulatory blood pressure in metabolic syndrome: a randomized SYSDIET sub-study
60	Brown, A. L.; Lane, J.; Coverly, J.; Stocks, J.; Jackson, S.; Stephen, A.; Bluck, L.; Coward, A.; Hendrickx, H.	2009	Effects of dietary supplementation with the green tea polyphenol epigallocatechin-3-gallate on insulin resistance and associated metabolic risk factors: randomized controlled trial
61	Cabello-Saavedra, E.; Bes-Rastrollo, M.; Martinez, J. A.; Diez-Espino, J.; Buil-Cosiales, P.; Serrano-Martinez, M.; Martinez-Gonzalez, M. A.	2010	Macronutrient intake and metabolic syndrome in subjects at high cardiovascular risk
62	Canhada, S. L.; Vigo, Á; Luft, V. C.; Levy, R. B.; Alvim Matos, S. M.; Del Carmen Molina, M.; Giatti, L.; Barreto, S.; Duncan, B. B.; Schmidt, M. I.	2023	Ultra-Processed Food Consumption and Increased Risk of Metabolic Syndrome in Adults: The ELSA-Brasil
63	Carlson, J. J.; Eisenmann, J. C.; Norman, G. J.; Ortiz, K. A.; Young, P. C.	2011	Dietary fiber and nutrient density are inversely associated with the metabolic syndrome in US adolescents
64	Carnethon, M. R.; Loria, C. M.; Hill, J. O.;	2004	Risk factors for the metabolic syndrome: the Coronary Artery Risk Development in Young Adults (CARDIA) study, 1985-2001

	Sidney, S.; Savage, P. J.; Liu, K.		
65	Castellino, G.; Nikolic, D.; Magán-Fernández, A.; Malfa, G. A.; Chianetta, R.; Patti, A. M.; Amato, A.; Montalto, G.; Toth, P. P.; Banach, M.; Cicero, A. F. G.; Rizzo, M.	2019	Altilix(®) Supplement Containing Chlorogenic Acid and Luteolin Improved Hepatic and Cardiometabolic Parameters in Subjects with Metabolic Syndrome: A 6 Month Randomized, Double-Blind, Placebo-Controlled Study
66	Castro-Barquero, S.; Tresserra-Rimbau, A.; Vitelli-Storelli, F.; Doménech, M.; Salas-Salvadó, J.; Martín-Sánchez, V.; Rubín-García, M.; Buil-Cosiales, P.; Corella, D.; Fitó, M.; Romaguera, D.; Vioque, J.; Alonso-Gómez Á, M.; Wärnberg, J.; Martínez, J. A.; Serra-Majem, L.; Tinahones, F. J.; Lapetra, J.; Pintó, X.; Tur, J. A.; Garcia-Rios, A.; García-Molina, L.; Delgado-Rodriguez, M.; Matía-Martín, P.; Daimiel, L.; Vidal, J.; Vázquez, C.; Cofán, M.; Romanos-Nanclares, A.; Becerra-Tomas, N.; Barragan, R.; Castañer, O.; Konieczna, J.; González-Palacios, S.; Sorto-Sánchez, C.; Pérez-López, J.; Zulet, M. A.; Bautista-Castaño, I.; Casas, R.; Gómez-Perez, A. M.; Santos-Lozano, J. M.; Rodríguez-Sanchez, MÁ; Julibert, A.; Martín-Calvo, N.; Hernández-Alonso, P.; Sorlí, J. V.; Sanllorente, A.; Galmés-Panadés, A. M.; Cases-Pérez, E.; Goicolea-Güemez, L.;	2020	Dietary Polyphenol Intake is Associated with HDL-Cholesterol and A Better Profile of other Components of the Metabolic Syndrome: A PREDIMED-Plus Sub-Study

	Ruiz-Canela, M.; Babio, N.; Hernáez, Á; Lamuela-Raventós, R. M.; Estruch, R.		
67	Chen, J.; Gu, D.; Huang, J.; Rao, D. C.; Jaquish, C. E.; Hixson, J. E.; Chen, C. S.; Chen, J.; Lu, F.; Hu, D.; Rice, T.; Kelly, T. N.; Hamm, L. L.; Whelton, P. K.; He, J.	2009	Metabolic syndrome and salt sensitivity of blood pressure in non-diabetic people in China: a dietary intervention study
68	Chen, X.; Pang, Z.; Li, K.	2009	Dietary fat, sedentary behaviors and the prevalence of the metabolic syndrome among Qingdao adults
69	Chen, Y.; Feng, R.; Yang, X.; Dai, J.; Huang, M.; Ji, X.; Li, Y.; Okekunle, A. P.; Gao, G.; Onwuka, J. U.; Pang, X.; Wang, C.; Li, C.; Li, Y.; Sun, C.	2019	Yogurt improves insulin resistance and liver fat in obese women with nonalcoholic fatty liver disease and metabolic syndrome: a randomized controlled trial
70	Chien, K. L.; Chao, C. L.; Kuo, C. H.; Lin, H. J.; Liu, P. H.; Chen, P. R.; Hsu, H. C.; Lee, B. C.; Lee, Y. T.; Chen, M. F.	2011	Plasma fatty acids and the risk of metabolic syndrome in ethnic Chinese adults in Taiwan
71	Choi, A.; Ha, K.; Joung, H.; Song, Y.	2019	Frequency of Consumption of Whole Fruit, Not Fruit Juice, Is Associated with Reduced Prevalence of Obesity in Korean Adults
72	Choi, M. K.; Bae, Y. J.	2013	Relationship between dietary magnesium, manganese, and copper and metabolic syndrome risk in Korean adults: the Korea National Health and Nutrition Examination Survey (2007-2008)
73	Chung, H. K.; Kim, J. H.; Choi, A.; Ahn, C. W.; Kim, Y. S.; Nam, J. S.	2022	Antioxidant-Rich Dietary Intervention Improves Cardiometabolic Profiles and Arterial Stiffness in Elderly Koreans with Metabolic Syndrome
74	Chung, S.; Ha, K.; Lee, H. S.; Kim, C. I.; Joung, H.; Paik, H. Y.; Song, Y.	2015	Soft drink consumption is positively associated with metabolic syndrome risk factors only in Korean women: Data from the 2007-2011 Korea National Health and Nutrition Examination Survey
75	Cicero, A. F.; Derosa, G.; Di Gregori, V.; Bove, M.; Gaddi, A. V.; Borghi, C.	2010	Omega 3 polyunsaturated fatty acids supplementation and blood pressure levels in hypertriglyceridemic patients with untreated normal-high blood pressure and with or without metabolic syndrome: a retrospective study

76	Cicero, A. F. G.; Fogacci, F.; Bove, M.; Giovannini, M.; Borghi, C.	2021	Impact of a short-term synbiotic supplementation on metabolic syndrome and systemic inflammation in elderly patients: a randomized placebo-controlled clinical trial
77	Cione, J. G. C.; Verlengia, R.; Barbosa, C. G. R.; Ribeiro, Agsv; de Oliveira, J. J.; Oliveira, M. A.; Crisp, A. H.	2021	No additional effects of ursolic acid supplementation associated with combined exercise program on metabolic syndrome of postmenopausal women: A double-blind, randomized, placebo-controlled trial
78	Citarrella, R.; Chianetta, R.; Amodeo, S.; Mirarchi, L.; Licata, A.; Soresi, M.; Veronese, N.; Barbagallo, M.; Giannitrapani, L.	2024	Effectiveness of a Food Supplement Based on Glucomannan, D-Chiro-Inositol, Cinnamomum zeylanicum Blume and Inulin in Patients with Metabolic Syndrome
79	Clark, R. L.; Famodu, O. A.; Holásková, I.; Infante, A. M.; Murray, P. J.; Olfert, I. M.; McFadden, J. W.; Downes, M. T.; Chantler, P. D.; Duespohl, M. W.; Cuff, C. F.; Olfert, M. D.	2019	Educational intervention improves fruit and vegetable intake in young adults with metabolic syndrome components
80	Cocate, P. G.; Natali, A. J.; de Oliveira, A.; Alfenas Rde, C.; Peluzio Mdo, C.; Longo, G. Z.; dos Santos, E. C.; Buthers, J. M.; de Oliveira, L. L.; Hermsdorff, H. H.	2015	Red but not white meat consumption is associated with metabolic syndrome, insulin resistance and lipid peroxidation in Brazilian middle-aged men
81	Crichton, G.; Alkerwi, A.; Elias, M.	2015	Diet Soft Drink Consumption is Associated with the Metabolic Syndrome: A Two Sample Comparison
82	Cubas-Basterrechea, G.; Elío, I.; Sumalla-Cano, S.; Aparicio-Obregón, S.; González-Antón, C. T.; Muñoz-Cacho, P.	2022	The Regular Consumption of Nuts Is Associated with a Lower Prevalence of Abdominal Obesity and Metabolic Syndrome in Older People from the North of Spain
83	Curtis, P. J.; van der Velpen, V.; Berends, L.; Jennings, A.; Feelisch, M.; Umpleby, A. M.; Evans, M.; Fernandez, B. O.; Meiss, M. S.; Minnion, M.; Potter, J.; Minihane, A. M.; Kay,	2019	Blueberries improve biomarkers of cardiometabolic function in participants with metabolic syndrome-results from a 6-month, double-blind, randomized controlled trial

	C. D.; Rimm, E. B.; Cassidy, A.		
84	Czernichow, S.; Vergnaud, A. C.; Galan, P.; Arnaud, J.; Favier, A.; Faure, H.; Huxley, R.; Hercberg, S.; Ahluwalia, N.	2009	Effects of long-term antioxidant supplementation and association of serum antioxidant concentrations with risk of metabolic syndrome in adults
85	Dall'Alba, V.; Silva, F. M.; Antonio, J. P.; Steemburgo, T.; Royer, C. P.; Almeida, J. C.; Gross, J. L.; Azevedo, M. J.	2013	Improvement of the metabolic syndrome profile by soluble fibre - guar gum - in patients with type 2 diabetes: a randomised clinical trial
86	Damsgaard, C. T.; Stark, K. D.; Hjorth, M. F.; Biltoft-Jensen, A.; Astrup, A.; Michaelsen, K. F.; Lauritzen, L.	2013	-3 PUFA status in school children is associated with beneficial lipid profile, reduced physical activity and increased blood pressure in boys
87	de Bock, M.; Derraik, J. G.; Brennan, C. M.; Biggs, J. B.; Smith, G. C.; Cameron-Smith, D.; Wall, C. R.; Cutfield, W. S.	2012	Psyllium supplementation in adolescents improves fat distribution & lipid profile: a randomized, participant-blinded, placebo-controlled, crossover trial
88	de la Iglesia, R.; Lopez-Legarrea, P.; Celada, P.; Sánchez-Muniz, F. J.; Martinez, J. A.; Zulet, M. A.	2013	Beneficial effects of the RESMENA dietary pattern on oxidative stress in patients suffering from metabolic syndrome with hyperglycemia are associated to dietary TAC and fruit consumption
89	de Mello Fontanelli, M.; Sales, C. H.; Carioca, A. A. F.; Marchioni, D. M.; Fisberg, R. M.	2018	The relationship between carbohydrate quality and the prevalence of metabolic syndrome: challenges of glycemic index and glycemic load
90	de Oliveira, E. P.; McLellan, K. C.; Vaz de Arruda Silveira, L.; Burini, R. C.	2012	Dietary factors associated with metabolic syndrome in Brazilian adults
91	de Oliveira Otto, M. C.; Alonso, A.; Lee, D. H.; Delclos, G. L.; Bertoni, A. G.; Jiang, R.; Lima, J. A.; Symanski, E.; Jacobs, D. R., Jr.; Nettleton, J. A.	2012	Dietary intakes of zinc and heme iron from red meat, but not from other sources, are associated with greater risk of metabolic syndrome and cardiovascular disease
92	de Souza Zanchet, M. Z.; Nardi, G. M.; de Oliveira Souza Bratti,	2017	Lycium barbarum Reduces Abdominal Fat and Improves Lipid Profile and Antioxidant Status in Patients with Metabolic Syndrome

	L.; Filippin-Monteiro, F. B.; Locatelli, C.		
93	Delavar, M. A.; Lye, M. S.; Khor, G. L.; Hassan, S. T.; Hanachi, P.	2009	Dietary patterns and the metabolic syndrome in middle aged women, Babol, Iran
94	Denova-Gutiérrez, E.; Talavera, J. O.; Huitrón-Bravo, G.; Méndez-Hernández, P.; Salmerón, J.	2010	Sweetened beverage consumption and increased risk of metabolic syndrome in Mexican adults
95	Desai, T.; Roberts, M.; Bottoms, L.	2021	Effects of short-term continuous Montmorency tart cherry juice supplementation in participants with metabolic syndrome
96	Deshmukh-Taskar, P.; Nicklas, T. A.; Radcliffe, J. D.; O'Neil, C. E.; Liu, Y.	2013	The relationship of breakfast skipping and type of breakfast consumed with overweight/obesity, abdominal obesity, other cardiometabolic risk factors and the metabolic syndrome in young adults. The National Health and Nutrition Examination Survey (NHANES): 1999-2006
97	Devaraj, S.; Yimam, M.; Brownell, L. A.; Jialal, I.; Singh, S.; Jia, Q.	2013	Effects of Aloe vera supplementation in subjects with prediabetes/metabolic syndrome
98	Dewell, A.; Marvasti, F. F.; Harris, W. S.; Tsao, P.; Gardner, C. D.	2011	Low- and high-dose plant and marine (n-3) fatty acids do not affect plasma inflammatory markers in adults with metabolic syndrome
99	Dhingra, R.; Sullivan, L.; Jacques, P. F.; Wang, T. J.; Fox, C. S.; Meigs, J. B.; D'Agostino, R. B.; Gaziano, J. M.; Vasan, R. S.	2007	Soft drink consumption and risk of developing cardiometabolic risk factors and the metabolic syndrome in middle-aged adults in the community
100	Díaz-Flores, M.; Cruz, M.; Duran-Reyes, G.; Munguia-Miranda, C.; Loza-Rodríguez, H.; Pulido-Casas, E.; Torres-Ramírez, N.; Gaja-Rodriguez, O.; Kumate, J.; Baiza-Gutman, L. A.; Hernández-Saavedra, D.	2013	Oral supplementation with glycine reduces oxidative stress in patients with metabolic syndrome, improving their systolic blood pressure
101	DiBello, J. R.; McGarvey, S. T.; Kraft, P.; Goldberg, R.; Campos, H.; Quested, C.; Laumoli, T. S.; Baylin, A.	2009	Dietary patterns are associated with metabolic syndrome in adult Samoans

102	Djoussé, L.; Arnett, D. K.; Eckfeldt, J. H.; Province, M. A.; Singer, M. R.; Ellison, R. C.	2004	Alcohol consumption and metabolic syndrome: does the type of beverage matter?
103	Dong, X. X.; Wang, R. R.; Liu, J. Y.; Ma, Q. H.; Pan, C. W.	2021	Habitual tea consumption and 5-year incident metabolic syndrome among older adults: a community-based cohort study
104	Dos Santos, M. C.; de Castro Coutinho, A. P. C.; de Souza Dantas, M.; Yabunaka, L. A. M.; Guedes, D. P.; Oesterreich, S. A.	2018	Correlates of metabolic syndrome among young Brazilian adolescents population
105	Driessen, M. T.; Koppes, L. L.; Veldhuis, L.; Samoocha, D.; Twisk, J. W.	2009	Coffee consumption is not related to the metabolic syndrome at the age of 36 years: the Amsterdam Growth and Health Longitudinal Study
106	Drouin-Chartier, J. P.; Gagnon, J.; Labonté, MÈ; Desroches, S.; Charest, A.; Grenier, G.; Dodin, S.; Lemieux, S.; Couture, P.; Lamarche, B.	2015	Impact of milk consumption on cardiometabolic risk in postmenopausal women with abdominal obesity
107	Duffey, K. J.; Gordon-Larsen, P.; Steffen, L. M.; Jacobs, D. R., Jr.; Popkin, B. M.	2010	Drinking caloric beverages increases the risk of adverse cardiometabolic outcomes in the Coronary Artery Risk Development in Young Adults (CARDIA) Study
108	Duffey, K. J.; Steffen, L. M.; Van Horn, L.; Jacobs, D. R., Jr.; Popkin, B. M.	2012	Dietary patterns matter: diet beverages and cardiometabolic risks in the longitudinal Coronary Artery Risk Development in Young Adults (CARDIA) Study
109	Dustin, D.; Kowalski, C.; Salesses, M.; McDowell, A.; Kris-Etherton, P. M.; Belury, M.; Johnson, L. K.; Conrad, Z.	2023	Carbohydrate Intakes Below Recommendations With a High Intake of Fat Are Associated With Higher Prevalence of Metabolic Syndrome
110	Ebrahimi, M.; Ghayour-Mobarhan, M.; Rezaiean, S.; Hoseini, M.; Parizade, S. M.; Farhoudi, F.; Hosseininezhad, S. J.; Tavallaei, S.; Vejdani, A.; Azimi-Nezhad, M.; Shakeri, M. T.; Rad, M. A.; Mobarra, N.; Kazemi-Bajestani, S. M.; Ferns, G. A.	2009	Omega-3 fatty acid supplements improve the cardiovascular risk profile of subjects with metabolic syndrome, including markers of inflammation and auto-immunity

111	Eilat-Adar, S.; Xu, J.; Goldbourt, U.; Zephier, E.; Howard, B. V.; Resnick, H. E.	2008	Sex may modify the effects of macronutrient intake on metabolic syndrome and insulin resistance in American Indians: the strong heart study
112	El Bilbeisi, A. H.; Hosseini, S.; Djafarian, K.	2017	Dietary Patterns and Metabolic Syndrome among Type 2 Diabetes Patients in Gaza Strip, Palestine
113	El-Sayed, E. F.; Awadalla, H.; Noor, S. K.; Elmadhoun, W. M.; Sulaiman, A. A.; Almobarak, A. O.; Ahmed, M. H.	2018	Sugar intake in Sudanese individuals was associated with some features of the metabolic syndrome: Population based study
114	Esfandiar, Z.; Hosseini-Esfahani, F.; Mirmiran, P.; Habibi-Moeini, A. S.; Azizi, F.	2019	Red meat and dietary iron intakes are associated with some components of metabolic syndrome: Tehran Lipid and Glucose Study
115	Esmaillzadeh, A.; Azadbakht, L.	2008	Consumption of hydrogenated versus nonhydrogenated vegetable oils and risk of insulin resistance and the metabolic syndrome among Iranian adult women
116	Esmaillzadeh, A.; Kimiagar, M.; Mehrabi, Y.; Azadbakht, L.; Hu, F. B.; Willett, W. C.	2006	Fruit and vegetable intakes, C-reactive protein, and the metabolic syndrome
117	Esmaillzadeh, A.; Kimiagar, M.; Mehrabi, Y.; Azadbakht, L.; Hu, F. B.; Willett, W. C.	2007	Dietary patterns, insulin resistance, and prevalence of the metabolic syndrome in women
118	Esmaillzadeh, A.; Mirmiran, P.; Azizi, F.	2005	Whole-grain consumption and the metabolic syndrome: a favorable association in Tehranian adults
119	Farag, H. A. M.; Hosseinzadeh-Attar, M. J.; Muhammad, B. A.; Esmaillzadeh, A.; Hamid El Bilbeisi, A.	2019	Effects of vitamin D supplementation along with endurance physical activity on lipid profile in metabolic syndrome patients: A randomized controlled trial
120	Farhat, S.; Zafar, M. U.; Sheikh, M. A.; Qasim, C. M.; Urooj, F.; Fatima, S. S.	2020	Association of resolvin level in pregnant women with preeclampsia and metabolic syndrome
121	Feng, R. N.; Niu, Y. C.; Sun, X. W.; Li, Q.; Zhao, C.; Wang, C.; Guo, F. C.; Sun, C. H.; Li, Y.	2013	Histidine supplementation improves insulin resistance through suppressed inflammation in obese women with the metabolic syndrome: a randomised controlled trial
122	Fernandes, R. R.; Nabuco, H. C. G.; Sugihara Junior, P.; Cavalcante, E. F.; Fabro, P. M. C.;	2018	Effect of protein intake beyond habitual intakes following resistance training on cardiometabolic risk disease parameters in pre-conditioned older women

	Tomeleri, C. M.; Ribeiro, A. S.; Barbosa, D. S.; Venturini, D.; Schoenfeld, B. J.; Cyrino, E. S.		
123	Fernández-Montero, A.; Bes-Rastrollo, M.; Beunza, J. J.; Barrio-Lopez, M. T.; de la Fuente-Arrillaga, C.; Moreno-Galarraga, L.; Martínez-González, M. A.	2013	Nut consumption and incidence of metabolic syndrome after 6-year follow-up: the SUN (Seguimiento Universidad de Navarra, University of Navarra Follow-up) cohort
124	Ferreira, P. P.; Cangussu, L.; Bueloni-Dias, F. N.; Orsatti, C. L.; Schmitt, E. B.; Nahas-Neto, J.; Nahas, E. A. P.	2020	Vitamin D supplementation improves the metabolic syndrome risk profile in postmenopausal women
125	Ferreira-Pêgo, C.; Babio, N.; Bes-Rastrollo, M.; Corella, D.; Estruch, R.; Ros, E.; Fitó, M.; Serra-Majem, L.; Arós, F.; Fiol, M.; Santos-Lozano, J. M.; Muñoz-Bravo, C.; Pintó, X.; Ruiz-Canela, M.; Salas-Salvadó, J.	2016	Frequent Consumption of Sugar- and Artificially Sweetened Beverages and Natural and Bottled Fruit Juices Is Associated with an Increased Risk of Metabolic Syndrome in a Mediterranean Population at High Cardiovascular Disease Risk
126	Flor-Alemany, M.; Acosta, P.; Marín-Jiménez, N.; Baena-García, L.; Aranda, P.; Aparicio, V. A.	2021	Influence of the degree of adherence to the mediterranean diet and its components on cardiometabolic risk during pregnancy. The GESTAFIT project
127	Ford, E. S.; Mokdad, A. H.; Giles, W. H.; Brown, D. W.	2003	The metabolic syndrome and antioxidant concentrations: findings from the Third National Health and Nutrition Examination Survey
128	Freire, R. D.; Cardoso, M. A.; Gimeno, S. G.; Ferreira, S. R.	2005	Dietary fat is associated with metabolic syndrome in Japanese Brazilians
129	Fujii, H.; Iwase, M.; Ohkuma, T.; Ogata-Kaizu, S.; Ide, H.; Kikuchi, Y.; Idewaki, Y.; Joudai, T.; Hirakawa, Y.; Uchida, K.; Sasaki, S.; Nakamura, U.; Kitazono, T.	2013	Impact of dietary fiber intake on glycemic control, cardiovascular risk factors and chronic kidney disease in Japanese patients with type 2 diabetes mellitus: the Fukuoka Diabetes Registry

130	Fujioka, K.; Greenway, F.; Sheard, J.; Ying, Y.	2006	The effects of grapefruit on weight and insulin resistance: relationship to the metabolic syndrome
131	Fulgoni, V. L., 3rd; Brauchla, M.; Fleige, L.; Chu, Y.	2020	Association of whole-grain and dietary fiber intake with cardiometabolic risk in children and adolescents
132	Fulgoni, V. L., 3rd; Dreher, M.; Davenport, A. J.	2013	Avocado consumption is associated with better diet quality and nutrient intake, and lower metabolic syndrome risk in US adults: results from the National Health and Nutrition Examination Survey (NHANES) 2001-2008
133	Fung, G. J.; Steffen, L. M.; Zhou, X.; Harnack, L.; Tang, W.; Lutsey, P. L.; Loria, C. M.; Reis, J. P.; Van Horn, L. V.	2012	Vitamin D intake is inversely related to risk of developing metabolic syndrome in African American and white men and women over 20 y: the Coronary Artery Risk Development in Young Adults study
134	Galié, S.; García-Gavilán, J.; Papandreou, C.; Camacho-Barcía, L.; Arcelin, P.; Palau-Galindo, A.; Rabassa, A.; Bulló, M.	2021	Effects of Mediterranean Diet on plasma metabolites and their relationship with insulin resistance and gut microbiota composition in a crossover randomized clinical trial
135	Galletti, F.; Fazio, V.; Gentile, M.; Schillaci, G.; Pucci, G.; Battista, F.; Mercurio, V.; Bosso, G.; Bonaduce, D.; Brambilla, N.; Vitalini, C.; D'Amato, M.; Giacovelli, G.	2019	Efficacy of a nutraceutical combination on lipid metabolism in patients with metabolic syndrome: a multicenter, double blind, randomized, placebo controlled trial
136	Ge, Z.; Guo, X.; Chen, X.; Tang, J.; Yan, L.; Ren, J.; Zhang, J.; Lu, Z.; Dong, J.; Xu, J.; Cai, X.; Liang, H.; Ma, J.	2015	Association between 24 h urinary sodium and potassium excretion and the metabolic syndrome in Chinese adults: the Shandong and Ministry of Health Action on Salt and Hypertension (SMASH) study
137	Gemeda, D.; Abebe, E.; Duguma, A.	2022	Metabolic Syndrome and Its Associated Factors among Type 2 Diabetic Patients in Southwest Ethiopia, 2021/2022
138	Georgoulis, M.; Kontogianni, M. D.; Margariti, A.; Tiniakos, D.; Fragopoulou, E.; Zafiropoulou, R.; Papatheodoridis, G.	2015	Associations between dietary intake and the presence of the metabolic syndrome in patients with non-alcoholic fatty liver disease
139	Georgoulis, M.; Yiannakouris, N.; Kechribari, I.; Lamprou, K.; Perraki, E.; Vagiakis, E.; Kontogianni, M. D.	2020	Cardiometabolic Benefits of a Weight-Loss Mediterranean Diet/Lifestyle Intervention in Patients with Obstructive Sleep Apnea: The "MIMOSA" Randomized Clinical Trial

140	Ghosn, B.; Falahi, E.; Keshteli, A. H.; Yazdannik, A. R.; Azadbakht, L.; Esmaillzadeh, A.	2021	Lack of association between nuts and legumes consumption and metabolic syndrome in young Iranian nurses
141	Giacco, R.; Lappi, J.; Costabile, G.; Kolehmainen, M.; Schwab, U.; Landberg, R.; Uusitupa, M.; Poutanen, K.; Pacini, G.; Rivellese, A. A.; Riccardi, G.; Mykkänen, H.	2013	Effects of rye and whole wheat versus refined cereal foods on metabolic risk factors: a randomised controlled two-centre intervention study
142	Gøbel, R. J.; Larsen, N.; Jakobsen, M.; Mølgaard, C.; Michaelsen, K. F.	2012	Probiotics to adolescents with obesity: effects on inflammation and metabolic syndrome
143	Godala, M.; Materek-Kuśmierkiewicz, I.; Moczulski, D.; Rutkowski, M.; Szatko, F.; Gaszyńska, E.; Tokarski, S.; Kowalski, J.	2017	The risk of plasma vitamin A, C, E and D deficiency in patients with metabolic syndrome: A case-control study
144	Godala, M. M.; Materek-Kuśmierkiewicz, I.; Moczulski, D.; Rutkowski, M.; Szatko, F.; Gaszyńska, E.; Tokarski, S.; Kowalski, J.	2016	Lower Plasma Levels of Antioxidant Vitamins in Patients with Metabolic Syndrome: A Case Control Study
145	Gomes, J. M. G.; Costa, J. D. A.; Alfenas, R. C. G.	2018	Effect of increased calcium consumption from fat-free milk in an energy-restricted diet on the metabolic syndrome and cardiometabolic outcomes in adults with type 2 diabetes mellitus: a randomised cross-over clinical trial
146	González-Palacios, S.; Oncina-Cánovas, A.; García-de-la-Hera, M.; Martínez-González, MÁ; Salas-Salvadó, J.; Corella, D.; Schröder, H.; Martínez, J. A.; Alonso-Gómez Á, M.; Wärnberg, J.; Romaguera, D.; López-Miranda, J.; Estruch, R.; Tinahones, F. J.; Lapetra, J.; Serra-	2023	Increased ultra-processed food consumption is associated with worsening of cardiometabolic risk factors in adults with metabolic syndrome: Longitudinal analysis from a randomized trial

	Majem, J. L.; Cano-Ibañez, N.; Tur, J. A.; Martín-Sánchez, V.; Pintó, X.; Delgado-Rodríguez, M.; Matía-Martín, P.; Vidal, J.; Vázquez, C.; Daimiel, L.; Ros, E.; Bes-Rastrollo, M.; Atzeni, A.; Sorli, J. V.; Zomeño, M. D.; Peña-Orihuela, P. J.; Compañ-Gabucio, L. M.; Barón-López, F. J.; Zulet, MÁ; Konieczna, J.; Casas, R. M.; Garrido-Garrido, E. M.; Tojal-Sierra, L.; Gomez-Perez, A. M.; Ruiz-Canela, M.; Palau, A.; Saiz, C.; Pérez-Vega, K. A.; Garcia-Rios, A.; Torres-Collado, L.; Basterra-Gortari, J.; Garcidueñas-Fimbres, T. E.; Malcampo, M.; Vioque, J.		
147	Gouni-Berthold, I.; Schulte, D. M.; Krone, W.; Lapointe, J. F.; Lemieux, P.; Predel, H. G.; Berthold, H. K.	2012	The whey fermentation product malleable protein matrix decreases TAG concentrations in patients with the metabolic syndrome: a randomised placebo-controlled trial
148	Grosso, G.; Marventano, S.; Galvano, F.; Pajak, A.; Mistretta, A.	2014	Factors associated with metabolic syndrome in a mediterranean population: role of caffeinated beverages
149	Grunwald, T.; Fadia, S.; Bernstein, B.; Naliborski, M.; Wu, S.; Luca, F.	2017	Vitamin D supplementation, the metabolic syndrome and oxidative stress in obese children
150	Gulseth, H. L.; Gjelstad, I. M.; Tierney, A. C.; Shaw, D. I.; Helal, O.; Hees, A. M.; Delgado-Lista, J.; Leszczynska-Golabek, I.; Karlström, B.; Lovegrove, J.; Defoort, C.; Blaak, E. E.; Lopez-Miranda, J.; Dembinska-Kiec, A.; Risérus, U.; Roche, H.	2010	Dietary fat modifications and blood pressure in subjects with the metabolic syndrome in the LIPGENE dietary intervention study

	M.; Birkeland, K. I.; Drevon, C. A.		
151	Guo, Z.; Miura, K.; Turin, T. C.; Hozawa, A.; Okuda, N.; Okamura, T.; Saitoh, S.; Sakata, K.; Nakagawa, H.; Okayama, A.; Yoshita, K.; Kadowaki, T.; Choudhury, S. R.; Nakamura, Y.; Rodriguez, B. L.; Curb, D. J.; Elliott, P.; Stamler, J.; Ueshima, H.	2010	Relationship of the polyunsaturated to saturated fatty acid ratio to cardiovascular risk factors and metabolic syndrome in Japanese: the INTERLIPID study
152	Harris Jackson, K.; West, S. G.; Vanden Heuvel, J. P.; Jonnalagadda, S. S.; Ross, A. B.; Hill, A. M.; Grieger, J. A.; Lemieux, S. K.; Kris-Etherton, P. M.	2014	Effects of whole and refined grains in a weight-loss diet on markers of metabolic syndrome in individuals with increased waist circumference: a randomized controlled-feeding trial
153	Hashimoto, Y.; Tanaka, M.; Miki, A.; Kobayashi, Y.; Wada, S.; Kuwahata, M.; Kido, Y.; Yamazaki, M.; Fukui, M.	2018	Intake of Carbohydrate to Fiber Ratio Is a Useful Marker for Metabolic Syndrome in Patients with Type 2 Diabetes: A Cross-Sectional Study
154	Hassannejad, R.; Mohammadifard, N.; Kazemi, I.; Mansourian, M.; Sadeghi, M.; Roohafza, H.; Sarrafzadegan, N.	2019	Long-term nuts intake and metabolic syndrome: A 13-year longitudinal population-based study
155	Hautaniemi, E. J.; Tikkakoski, A. J.; Tahvanainen, A.; Nordhausen, K.; Kähönen, M.; Mattsson, T.; Luhtala, S.; Turpeinen, A. M.; Niemelä, O.; Vapaatalo, H.; Korpela, R.; Pörsti, I. H.	2015	Effect of fermented milk product containing lactotripeptides and plant sterol esters on haemodynamics in subjects with the metabolic syndrome--a randomised, double-blind, placebo-controlled study
156	Hekmatdoost, A.; Mirmiran, P.; Hosseini-Esfahani, F.; Azizi, F.	2011	Dietary fatty acid composition and metabolic syndrome in Tehranian adults

157	Hernández-Cordero, S.; Barquera, S.; Rodríguez-Ramírez, S.; Villanueva-Borbolla, M. A.; González de Cossio, T.; Dommarco, J. R.; Popkin, B.	2014	Substituting water for sugar-sweetened beverages reduces circulating triglycerides and the prevalence of metabolic syndrome in obese but not in overweight Mexican women in a randomized controlled trial
158	Heshmatipour, H.; Hajhashemy, Z.; Mirzaei, S.; Asadi, A.; Akhlaghi, M.; Saneei, P.	2023	Association of legumes and nuts consumption with metabolic health status in Iranian overweight and obese adolescents
159	Hess, E. L.; Myers, E. A.; Swithers, S. E.; Hedrick, V. E.	2018	Associations Between Nonnutritive Sweetener Intake and Metabolic Syndrome in Adults
160	Hino, A.; Adachi, H.; Enomoto, M.; Furuki, K.; Shigetoh, Y.; Ohtsuka, M.; Kumagae, S.; Hirai, Y.; Jalaldin, A.; Satoh, A.; Imaizumi, T.	2007	Habitual coffee but not green tea consumption is inversely associated with metabolic syndrome: an epidemiological study in a general Japanese population
161	Hoffmann, I. S.; Cubeddu, L. X.	2007	Increased blood pressure reactivity to dietary salt in patients with the metabolic syndrome
162	Hoffmann, I. S.; Cubeddu, L. X.	2009	Salt and the metabolic syndrome
163	Holligan, S. D.; West, S. G.; Gebauer, S. K.; Kay, C. D.; Kris-Etherton, P. M.	2014	A moderate-fat diet containing pistachios improves emerging markers of cardiometabolic syndrome in healthy adults with elevated LDL levels
164	Holt, E. W.; Wei, E. K.; Bennett, N.; Zhang, L. M.	2014	Low skin carotenoid concentration measured by resonance Raman spectroscopy is associated with metabolic syndrome in adults
165	Holthaus, T. A.; Sethi, S.; Cannavale, C. N.; Aguiñaga, S.; Burd, N. A.; Holscher, H. D.; Khan, N. A.	2023	MIND dietary pattern adherence is inversely associated with visceral adiposity and features of metabolic syndrome
166	Hong, S. A.; Kim, M. K.	2017	Relationship between fruit and vegetable intake and the risk of metabolic syndrome and its disorders in Korean women according to menopausal status
167	Hosseini-Esfahani, F.; Jessri, M.; Mirmiran, P.; Sadeghi, M.; Azizi, F.	2011	Does the diet of Tehranian adults ensure compliance with nutritional targets? Observations from the Tehran Lipid and Glucose Study
168	Hosseinpour-Niazi, S.; Aghayan, M.; Mirmiran, P.; Azizi, F.	2021	Does weight change modify the association between the consumption of sugar-sweetened beverages and 100% fruit juice and the risk of metabolic syndrome?

169	Hosseinpour-Niazi, S.; Bakhshi, B.; Mirmiran, P.; Azizi, F.	2021	Socioeconomic and lifestyle factors modifies the association between nut consumption and metabolic syndrome incidence
170	Hosseinpour-Niazi, S.; Mirmiran, P.; Fallah-ghohroudi, A.; Azizi, F.	2015	Combined effect of unsaturated fatty acids and saturated fatty acids on the metabolic syndrome: Tehran lipid and glucose study
171	Høstmark, A. T.	2010	The Oslo Health Study: a Dietary Index estimating high intake of soft drinks and low intake of fruits and vegetables was positively associated with components of the metabolic syndrome
172	Høstmark, A. T.	2010	The Oslo health study: soft drink intake is associated with the metabolic syndrome
173	Huang, L.; Wang, H.; Wang, Z.; Zhang, J.; Zhang, B.; Ding, G.	2019	Regional Disparities in the Association between Cereal Consumption and Metabolic Syndrome: Results from the China Health and Nutrition Survey
174	Huang, L. N.; Wang, H. J.; Wang, Z. H.; Zhang, J. G.; Jia, X. F.; Zhang, B.; Ding, G. Q.	2020	Association of Red Meat Usual Intake with Serum Ferritin and the Risk of Metabolic Syndrome in Chinese Adults: A Longitudinal Study from the China Health and Nutrition Survey
175	Huang, T.; Bhulaidok, S.; Cai, Z.; Xu, T.; Xu, F.; Wahlqvist, M. L.; Li, D.	2010	Plasma phospholipids n-3 polyunsaturated fatty acid is associated with metabolic syndrome
176	Hur, Y. I.; Park, H.; Kang, J. H.; Lee, H. A.; Song, H. J.; Lee, H. J.; Kim, O. H.	2015	Associations between Sugar Intake from Different Food Sources and Adiposity or Cardio-Metabolic Risk in Childhood and Adolescence: The Korean Child-Adolescent Cohort Study
177	Ibarrola-Jurado, N.; Bulló, M.; Guasch-Ferré, M.; Ros, E.; Martínez-González, M. A.; Corella, D.; Fiol, M.; Wärnberg, J.; Estruch, R.; Román, P.; Arós, F.; Vinyoles, E.; Serra-Majem, L.; Pintó, X.; Covas, M. I.; Basora, J.; Salas-Salvadó, J.	2013	Cross-sectional assessment of nut consumption and obesity, metabolic syndrome and other cardiometabolic risk factors: the PREDIMED study
178	Inoue, H.; Sasaki, R.; Aiso, I.; Kuwano, T.	2014	Short-term intake of a Japanese-style healthy lunch menu contributes to prevention and/or improvement in metabolic syndrome among middle-aged men: a non-randomized controlled trial
179	Iwasaki, Y.; Arisawa, K.; Katsuura-Kamano, S.; Uemura, H.; Tsukamoto, M.;	2019	Associations of Nutrient Patterns with the Prevalence of Metabolic Syndrome: Results from the Baseline Data of the Japan Multi-Institutional Collaborative Cohort Study

	Kadomatsu, Y.; Okada, R.; Hishida, A.; Tanaka, K.; Hara, M.; Takezaki, T.; Shimatani, K.; Ozaki, E.; Koyama, T.; Suzuki, S.; Nakagawa-Senda, H.; Kuriki, K.; Miyagawa, N.; Kadota, A.; Ikezaki, H.; Furusyo, N.; Oze, I.; Ito, H.; Mikami, H.; Nakamura, Y.; Wakai, K.		
180	Izzo, R.; de Simone, G.; Giudice, R.; Chinali, M.; Trimarco, V.; De Luca, N.; Trimarco, B.	2010	Effects of nutraceuticals on prevalence of metabolic syndrome and on calculated Framingham Risk Score in individuals with dyslipidemia
181	Jääskeläinen, P.; Magnussen, C. G.; Pahkala, K.; Mikkilä, V.; Kähönen, M.; Sabin, M. A.; Fogelholm, M.; Hutri-Kähönen, N.; Taittonen, L.; Telama, R.; Laitinen, T.; Jokinen, E.; Lehtimäki, T.; Viikari, J. S.; Raitakari, O. T.; Juonala, M.	2012	Childhood nutrition in predicting metabolic syndrome in adults: the cardiovascular risk in Young Finns Study
182	Jaceldo-Siegl, K.; Haddad, E.; Oda, K.; Fraser, G. E.; Sabaté, J.	2014	Tree nuts are inversely associated with metabolic syndrome and obesity: the Adventist health study-2
183	Jarrar, A. H.; Stojanovska, L.; Apostolopoulos, V.; Feehan, J.; Bataineh, M. F.; Ismail, L. C.; Al Dhaheri, A. S.	2021	The Effect of Gum Arabic (Acacia Senegal) on Cardiovascular Risk Factors and Gastrointestinal Symptoms in Adults at Risk of Metabolic Syndrome: A Randomized Clinical Trial
184	Jiménez-Gómez, Y.; Marín, C.; Peérez-Martínez, P.; Hartwich, J.; Malczewska-Malec, M.; Golabek, I.; Kiec-Wilk, B.; Cruz-Teno, C.; Rodríguez, F.; Gómez, P.; Gómez-Luna, M. J.; Defoort, C.; Gibney, M. J.; Pérez-Jiménez, F.; Roche, H. M.; López-Miranda, J.	2010	A low-fat, high-complex carbohydrate diet supplemented with long-chain (n-3) fatty acids alters the postprandial lipoprotein profile in patients with metabolic syndrome

185	Johnson, S. A.; Navaei, N.; Pourafshar, S.; Jaime, S. J.; Akhavan, N. S.; Alvarez-Alvarado, S.; Proaño, G. V.; Litwin, N. S.; Clark, E. A.; Foley, E. M.; George, K. S.; Elam, M. L.; Payton, M. E.; Arjmandi, B. H.; Figueroa, A.	2020	Effects of Montmorency Tart Cherry Juice Consumption on Cardiometabolic Biomarkers in Adults with Metabolic Syndrome: A Randomized Controlled Pilot Trial
186	Julibert, A.; Bibiloni, M. D. M.; Mateos, D.; Angullo, E.; Tur, J. A.	2019	Dietary Fat Intake and Metabolic Syndrome in Older Adults
187	Julibert, A.; Del Mar Bibiloni, M.; Gallardo-Alfaro, L.; Abbate, M.; Martínez-González, MÁ; Salas-Salvadó, J.; Corella, D.; Fitó, M.; Martínez, J. A.; Alonso-Gómez Á, M.; Wärnberg, J.; Vioque, J.; Romaguera, D.; Lopez-Miranda, J.; Estruch, R.; Tinahones, F. J.; Lapetra, J.; Serra-Majem, L.; Cano-Ibañez, N.; Martín-Sánchez, V.; Pintó, X.; Gaforio, J. J.; Matía-Martín, P.; Vidal, J.; Vázquez, C.; Daimiel, L.; Ros, E.; Sayon-Orea, C.; Becerra-Tomás, N.; Gimenez-Alba, I. M.; Castañer, O.; Abete, I.; Tojal-Sierra, L.; Pérez-López, J.; Notario-Barandiaran, L.; Colom, A.; Garcia-Rios, A.; Castro-Barquero, S.; Bernal, R.; Santos-Lozano, J. M.; Fernández-Lázaro, C. I.; Hernández-Alonso, P.; Saiz, C.; Zomeño, M. D.; Zulet, M. A.; Belló-Mora, M. C.; Basterra-Gortari, J.; Canudas, S.; Goday, A.; Tur, J. A.	2020	Metabolic Syndrome Features and Excess Weight Were Inversely Associated with Nut Consumption after 1-Year Follow-Up in the PREDIMED-Plus Study

188	Juna, C. F.; Cho, Y.; Ham, D.; Joung, H.	2021	Association of Carbohydrate and Fat Intake with Prevalence of Metabolic Syndrome Can Be Modified by Physical Activity and Physical Environment in Ecuadorian Adults: The ENSANUT-ECU Study
189	Jung, J. Y.; Park, S. K.; Oh, C. M.; Choi, J. M.; Ryoo, J. H.; Kim, J.; Kim, M. K.	2019	The association between metabolic syndrome and peanuts, pine nuts, almonds consumption: The Ansan and Ansung Study
190	Kamińska, S.; Pikala, M.; Dziankowska-Zaborszczyk, E.; Bielecki, W.; Rębowska, E.; Kozakiewicz, K.; Nadrowski, P.; Drygas, W.; Kwaśniewska, M.	2020	Vitamin D - dietary intake, supplementation and metabolic status of Polish adults
191	Kang, M. S.; Jang, K. A.; Kim, H. R.; Song, S.	2024	Association of Dietary Resistant Starch Intake with Obesity and Metabolic Syndrome in Korean Adults
192	Kang, Y.; Kim, J.	2016	Association between fried food consumption and hypertension in Korean adults
193	Kang, Y.; Kim, J.	2016	Gender difference on the association between dietary patterns and metabolic syndrome in Korean population
194	Kang, Y.; Kim, J.	2017	Soft drink consumption is associated with increased incidence of the metabolic syndrome only in women
195	Kang, Y.; Lee, K.; Lee, J.; Kim, J.	2020	Grain Subtype and the Combination of Grains Consumed Are Associated with the Risk of Metabolic Syndrome: Analysis of a Community-Based Prospective Cohort
196	Kassaian, N.; Feizi, A.; Aminorroaya, A.; Amini, M.	2019	Probiotic and synbiotic supplementation could improve metabolic syndrome in prediabetic adults: A randomized controlled trial
197	Katcher, H. I.; Legro, R. S.; Kunselman, A. R.; Gillies, P. J.; Demers, L. M.; Bagshaw, D. M.; Kris-Etherton, P. M.	2008	The effects of a whole grain-enriched hypocaloric diet on cardiovascular disease risk factors in men and women with metabolic syndrome
198	Kelishadi, R.; Gouya, M. M.; Adeli, K.; Ardalan, G.; Gheiratmand, R.; Majdzadeh, R.; Mahmoud-Arabi, M. S.; Delavari, A.; Riazi, M. M.; Barekati, H.; Motaghian, M.;	2008	Factors associated with the metabolic syndrome in a national sample of youths: CASPIAN Study

	Shariatinejad, K.; Heshmat, R.		
199	Kelishadi, R.; Salek, S.; Salek, M.; Hashemipour, M.; Movahedian, M.	2014	Effects of vitamin D supplementation on insulin resistance and cardiometabolic risk factors in children with metabolic syndrome: a triple-masked controlled trial
200	Kenig, S.; Kramberger, K.; Šik Novak, K.; Karnjuš, I.; Bandelj, D.; Petelin, A.; Jenko Pražnikar, Z.	2022	Helichrysum italicum (Roth) G. Don and Helichrysum arenarium (L.) Moench infusions in reversing the traits of metabolic syndrome: a double-blind randomized comparative trial
201	Khayyatzadeh, S. S.; Moohebati, M.; Mazidi, M.; Avan, A.; Tayefi, M.; Parizadeh, S. M.; Ebrahimi, M.; Heidari-Bakavoli, A.; Azarpazhooh, M. R.; Esmaily, H.; Ferns, G. A.; Nematy, M.; Safarian, M.; Ghayour-Mobarhan, M.	2016	Nutrient patterns and their relationship to metabolic syndrome in Iranian adults
202	Khosravi-Boroujeni, H.; Sarrafzadegan, N.; Mohammadifard, N.; Alikhasi, H.; Sajjadi, F.; Asgari, S.; Esmaillzadeh, A.	2012	Consumption of sugar-sweetened beverages in relation to the metabolic syndrome among Iranian adults
203	Kim, B. K.; Lim, Y. H.; Kim, S. G.; Kim, Y. M.; Shin, J.	2012	Relationship between sodium intake and blood pressure according to metabolic syndrome status in the Korean National Health and Nutrition Examination Survey
204	Kim, H.; Lee, K.; Rebholz, C. M.; Kim, J.	2021	Association between unhealthy plant-based diets and the metabolic syndrome in adult men and women: a population-based study in South Korea
205	Kim, H.; Simbo, S. Y.; Fang, C.; McAlister, L.; Roque, A.; Banerjee, N.; Talcott, S. T.; Zhao, H.; Kreider, R. B.; Mertens-Talcott, S. U.	2018	Açaí (Euterpe oleracea Mart.) beverage consumption improves biomarkers for inflammation but not glucose- or lipid-metabolism in individuals with metabolic syndrome in a randomized, double-blinded, placebo-controlled clinical trial
206	Kim, H. J.; Cho, S.; Jacobs, D. R., Jr.; Park, K.	2014	Instant coffee consumption may be associated with higher risk of metabolic syndrome in Korean adults
207	Kim, H. N.; Kim, S. H.; Eun, Y. M.; Song, S. W.	2018	Effects of zinc, magnesium, and chromium supplementation on cardiometabolic risk in adults with metabolic syndrome: A double-blind, placebo-controlled randomised trial

208	Kim, J.; Jo, I.	2011	Grains, vegetables, and fish dietary pattern is inversely associated with the risk of metabolic syndrome in South korean adults
209	Kim, J.; Tan, L. J.; Jung, H.; Roh, Y.; Lim, K.; Shin, S.	2022	The association between fruit and vegetable consumption and metabolic syndrome in Korean adults: does multivitamin use matter?
210	Kim, K.; Chang, Y.	2021	Association of dietary fiber intake with metabolic syndrome among adult cancer survivors: a population-based cross-sectional study
211	Kim, K.; Kim, K.; Park, S. M.	2016	Association between the Prevalence of Metabolic Syndrome and the Level of Coffee Consumption among Korean Women
212	Kim, R. J.; Wang, L.; Worley, S.; Leonard, D.	2018	Nut consumption and metabolic syndrome in US adolescents
213	Kim, S.; Song, Y.; Lee, J. E.; Jun, S.; Shin, S.; Wie, G. A.; Cho, Y. H.; Joung, H.	2017	Total Antioxidant Capacity from Dietary Supplement Decreases the Likelihood of Having Metabolic Syndrome in Korean Adults
214	Kim, S. A.; Shin, S.	2019	The Association between Coffee Consumption Pattern and Prevalence of Metabolic Syndrome in Korean Adults
215	Kim, T.; Kang, J.	2020	Association Between Serum Retinol and α-Tocopherol Levels and Metabolic Syndrome in Korean General Population: Analysis of Population-based Nationally Representative Data
216	Kim, Y.; Je, Y.	2018	Moderate coffee consumption is inversely associated with the metabolic syndrome in the Korean adult population
217	Kimokoti, R. W.; Gona, P.; Zhu, L.; Newby, P. K.; Millen, B. E.; Brown, L. S.; D'Agostino, R. B.; Fung, T. T.	2012	Dietary patterns of women are associated with incident abdominal obesity but not metabolic syndrome
218	Kjølbæk, L.; Benítez-Páez, A.; Gómez Del Pulgar, E. M.; Brahe, L. K.; Liebisch, G.; Matysik, S.; Rampelli, S.; Vermeiren, J.; Brigidi, P.; Larsen, L. H.; Astrup, A.; Sanz, Y.	2020	Arabinoxylan oligosaccharides and polyunsaturated fatty acid effects on gut microbiota and metabolic markers in overweight individuals with signs of metabolic syndrome: A randomized cross-over trial
219	Kofoed, C. L.; Christensen, J.; Dragsted, L. O.; Tjønneland, A.; Roswall, N.	2015	Determinants of dietary supplement use--healthy individuals use dietary supplements

220	Koite, N. L. N.; Sanogo, N. I.; Lépine, O.; Bard, J. M.; Ouguerram, K.	2022	Antioxidant Efficacy of a Spirulina Liquid Extract on Oxidative Stress Status and Metabolic Disturbances in Subjects with Metabolic Syndrome
221	Kokkinopoulou, A.; Pagkalos, I.; Rodopaios, N. E.; Koulouri, A. A.; Vasara, E.; Papadopoulou, S. K.; Skepastianos, P.; Hassapidou, M.; Kafatos, A. G.	2023	Does Religious Fasting Have a Protective Role against Metabolic Syndrome in Individuals Aged >50 Years?
222	Kong, J. S.; Lee, J.; Kim, Y.; Woo, H. W.; Shin, M. H.; Koh, S. B.; Kim, H. C.; Kim, Y. M.; Kim, M. K.	2023	Associations of cumulative average dietary total antioxidant capacity and intake of antioxidants with metabolic syndrome risk in Korean adults aged 40 years and older: a prospective cohort study (KoGES_CAVAS)
223	Kouki, R.; Schwab, U.; Hassinen, M.; Komulainen, P.; Heikkilä, H.; Lakka, T. A.; Rauramaa, R.	2011	Food consumption, nutrient intake and the risk of having metabolic syndrome: the DR's EXTRA Study
224	Koyama, T.; Maekawa, M.; Ozaki, E.; Kuriyama, N.; Uehara, R.	2020	Daily Consumption of Coffee and Eating Bread at Breakfast Time Is Associated with Lower Visceral Adipose Tissue and with Lower Prevalence of Both Visceral Obesity and Metabolic Syndrome in Japanese Populations: A Cross-Sectional Study
225	Krewer Cda, C.; Ribeiro, E. E.; Ribeiro, E. A.; Moresco, R. N.; da Rocha, M. I.; Montagner, G. F.; Machado, M. M.; Viegas, K.; Brito, E.; da Cruz, I. B.	2011	Habitual intake of guaraná and metabolic morbidities: an epidemiological study of an elderly Amazonian population
226	Kwon, H. T.; Lee, C. M.; Park, J. H.; Ko, J. A.; Seong, E. J.; Park, M. S.; Cho, B.	2010	Milk intake and its association with metabolic syndrome in Korean: analysis of the third Korea National Health and Nutrition Examination Survey (KNHANES III)
227	Kwon, O. W.; Jun, D. W.; Lee, S. M.; Lee, K. N.; Lee, H. L.; Lee, O. Y.; Yoon, B. C.; Choi, H. S.	2012	Carbohydrate but not fat is associated with elevated aminotransferases
228	Kwon, Y. J.; Lee, H. S.; Lee, J. W.	2018	Association of carbohydrate and fat intake with metabolic syndrome
229	Lai, Y. H.; Petrone, A. B.; Pankow, J. S.; Arnett, D. K.; North, K.	2013	Association of dietary omega-3 fatty acids with prevalence of metabolic syndrome: the National Heart, Lung, and Blood Institute Family Heart Study

	E.; Ellison, R. C.; Hunt, S. C.; Djoussé, L.		
230	Lakshmipriya, N.; Gayathri, R.; Praseena, K.; Vijayalakshmi, P.; Geetha, G.; Sudha, V.; Krishnaswamy, K.; Anjana, R. M.; Henry, J.; Mohan, V.	2013	Type of vegetable oils used in cooking and risk of metabolic syndrome among Asian Indians
231	Lankinen, M.; Kolehmainen, M.; Jääskeläinen, T.; Paananen, J.; Joukamo, L.; Kangas, A. J.; Soininen, P.; Poutanen, K.; Mykkänen, H.; Gylling, H.; Orešič, M.; Jauhiainen, M.; Ala-Korpela, M.; Uusitupa, M.; Schwab, U.	2014	Effects of whole grain, fish and bilberries on serum metabolic profile and lipid transfer protein activities: a randomized trial (Sysdimet)
232	Laso, N.; Brugué, E.; Vidal, J.; Ros, E.; Arnaiz, J. A.; Carné, X.; Vidal, S.; Mas, S.; Deulofeu, R.; Lafuente, A.	2007	Effects of milk supplementation with conjugated linoleic acid (isomers cis-9, trans-11 and trans-10, cis-12) on body composition and metabolic syndrome components
233	Laue, C.; Papazova, E.; Pannenbeckers, A.; Schrezenmeir, J.	2023	Effect of a Probiotic and a Synbiotic on Body Fat Mass, Body Weight and Traits of Metabolic Syndrome in Individuals with Abdominal Overweight: A Human, Double-Blind, Randomised, Controlled Clinical Study
234	Lauritzen, L.; Harsløf, L. B.; Hellgren, L. I.; Pedersen, M. H.; Mølgaard, C.; Michaelsen, K. F.	2012	Fish intake, erythrocyte n-3 fatty acid status and metabolic health in Danish adolescent girls and boys
235	Lawlor, D. A.; Ebrahim, S.; Timpson, N.; Davey Smith, G.	2005	Avoiding milk is associated with a reduced risk of insulin resistance and the metabolic syndrome: findings from the British Women's Heart and Health Study
236	Leão, Lscs; Aquino, L. A.; Dias, J. F.; Koifman, R. J.	2019	Addition of oat bran reduces HDL-C and does not potentialize effect of a low-calorie diet on remission of metabolic syndrome: A pragmatic, randomized, controlled, open-label nutritional trial
237	Lee, H.; Lee, J.; Hwang, S. S.; Kim, S.; Chin, H. J.; Han, J. S.; Heo, N. J.	2013	Potassium intake and the prevalence of metabolic syndrome: the Korean National Health and Nutrition Examination Survey 2008-2010

238	Lee, I. T.; Lee, W. J.; Tsai, C. M.; Su, I. J.; Yen, H. T.; Sheu, W. H.	2012	Combined extractives of red yeast rice, bitter gourd, chlorella, soy protein, and licorice improve total cholesterol, low-density lipoprotein cholesterol, and triglyceride in subjects with metabolic syndrome
239	Lee, K. W.; Cho, Y.; Jo, G.; Park, Y. K.; Shin, M. J.	2016	Association of dietary intakes of total and subtypes of fat substituted for carbohydrate with metabolic syndrome in Koreans
240	Lee, K. W.; Shin, D.	2018	A Healthy Beverage Consumption Pattern Is Inversely Associated with the Risk of Obesity and Metabolic Abnormalities in Korean Adults
241	Lee, S.; Shin, Y.; Kim, Y.	2018	Risk of Metabolic Syndrome among Middle-Aged Koreans from Rural and Urban Areas
242	Lee, Y. A.; Song, S. W.; Kim, S. H.; Kim, H. N.	2023	Associations between Dietary Patterns and Metabolic Syndrome: Findings of the Korean National Health and Nutrition Examination Survey
243	Lee, Y. J.; Nam, G. E.; Seo, J. A.; Yoon, T.; Seo, I.; Lee, J. H.; Im, D.; Bahn, K. N.; Jeong, S. A.; Kang, T. S.; Ahn, J. H.; Kim, D. H.; Kim, N. H.	2014	Nut consumption has favorable effects on lipid profiles of Korean women with metabolic syndrome
244	Lee, Y. J.; Seo, J. A.; Yoon, T.; Seo, I.; Lee, J. H.; Im, D.; Lee, J. H.; Bahn, K. N.; Ham, H. S.; Jeong, S. A.; Kang, T. S.; Ahn, J. H.; Kim, D. H.; Nam, G. E.; Kim, N. H.	2016	Effects of low-fat milk consumption on metabolic and atherogenic biomarkers in Korean adults with the metabolic syndrome: a randomised controlled trial
245	Lee, Y. J.; Song, S.; Song, Y.	2018	High-Carbohydrate Diets and Food Patterns and Their Associations with Metabolic Disease in the Korean Population
246	Leermakers, E. T.; Felix, J. F.; Jaddoe, V. W.; Raat, H.; Franco, O. H.; Kiefte-de Jong, J. C.	2015	Sugar-containing beverage intake at the age of 1 year and cardiometabolic health at the age of 6 years: the Generation R Study
247	Lehtonen, H. M.; Suomela, J. P.; Tahvonen, R.; Vaarno, J.; Venojärvi, M.; Viikari, J.; Kallio, H.	2010	Berry meals and risk factors associated with metabolic syndrome
248	Lehtonen, H. M.; Suomela, J. P.; Tahvonen, R.; Yang, B.; Venojärvi, M.; Viikari, J.; Kallio, H.	2011	Different berries and berry fractions have various but slightly positive effects on the associated variables of metabolic diseases on overweight and obese women

249	Lerman, R. H.; Minich, D. M.; Darland, G.; Lamb, J. J.; Chang, J. L.; Hsi, A.; Bland, J. S.; Tripp, M. L.	2010	Subjects with elevated LDL cholesterol and metabolic syndrome benefit from supplementation with soy protein, phytosterols, hops rho iso-alpha acids, and Acacia nilotica proanthocyanidins
250	Li, G.; de Courten, M.; Jiao, S.; Wang, Y.	2010	Prevalence and characteristics of the metabolic syndrome among adults in Beijing, China
251	Li, S.; Cao, M.; Yang, C.; Zheng, H.; Zhu, Y.	2020	Association of sugar-sweetened beverage intake with risk of metabolic syndrome among children and adolescents in urban China
252	Li, S.; Guerin-Deremaux, L.; Pochat, M.; Wils, D.; Reifer, C.; Miller, L. E.	2010	NUTRIOSE dietary fiber supplementation improves insulin resistance and determinants of metabolic syndrome in overweight men: a double-blind, randomized, placebo-controlled study
253	Li, X.; Guo, B.; Jin, D.; Wang, Y.; Jiang, Y.; Zhu, B.; Chen, Y.; Ma, L.; Zhou, H.; Xie, G.	2018	Association of dietary sodium:potassium ratio with the metabolic syndrome in Chinese adults
254	Li, X. T.; Liao, W.; Yu, H. J.; Liu, M. W.; Yuan, S.; Tang, B. W.; Yang, X. H.; Song, Y.; Huang, Y.; Cheng, S. L.; Chen, Z. Y.; Towne, S. D., Jr.; Mao, Z. F.; He, Q. Q.	2017	Combined effects of fruit and vegetables intake and physical activity on the risk of metabolic syndrome among Chinese adults
255	Liao, Z. Y.; Xiao, M. H.; She, Q.; Xiong, B. Q.	2024	Association between the composite dietary antioxidant index and metabolic syndrome: evidence from NHANES 2003-2018
256	Lie, L.; Brown, L.; Forrester, T. E.; Plange-Rhule, J.; Bovet, P.; Lambert, E. V.; Layden, B. T.; Luke, A.; Dugas, L. R.	2018	The Association of Dietary Fiber Intake with Cardiometabolic Risk in Four Countries across the Epidemiologic Transition
257	Lien, L. F.; Brown, A. J.; Ard, J. D.; Loria, C.; Erlinger, T. P.; Feldstein, A. C.; Lin, P. H.; Champagne, C. M.; King, A. C.; McGuire, H. L.; Stevens, V. J.; Brantley, P. J.; Harsha, D. W.; McBurnie, M. A.; Appel, L. J.; Svetkey, L. P.	2007	Effects of PREMIER lifestyle modifications on participants with and without the metabolic syndrome

258	Lim, M.; Kim, J.	2020	Association between fruit and vegetable consumption and risk of metabolic syndrome determined using the Korean Genome and Epidemiology Study (KoGES)
259	Lin, W. T.; Huang, H. L.; Huang, M. C.; Chan, T. F.; Ciou, S. Y.; Lee, C. Y.; Chiu, Y. W.; Duh, T. H.; Lin, P. L.; Wang, T. N.; Liu, T. Y.; Lee, C. H.	2013	Effects on uric acid, body mass index and blood pressure in adolescents of consuming beverages sweetened with high-fructose corn syrup
260	Liu, J.; Fulda, K. G.; Tao, M. H.	2021	Association between acculturation and metabolic syndrome in Hispanic adults mediated by fruits intake
261	Liu, S.; Song, Y.; Ford, E. S.; Manson, J. E.; Buring, J. E.; Ridker, P. M.	2005	Dietary calcium, vitamin D, and the prevalence of metabolic syndrome in middle-aged and older U.S. women
262	Liu, X.; Garban, J.; Jones, P. J.; Vanden Heuvel, J.; Lamarche, B.; Jenkins, D. J.; Connelly, P. W.; Couture, P.; Pu, S.; Fleming, J. A.; West, S. G.; Kris-Etherton, P. M.	2018	Diets Low in Saturated Fat with Different Unsaturated Fatty Acid Profiles Similarly Increase Serum-Mediated Cholesterol Efflux from THP-1 Macrophages in a Population with or at Risk for Metabolic Syndrome: The Canola Oil Multicenter Intervention Trial
263	Liu, X.; Kris-Etherton, P. M.; West, S. G.; Lamarche, B.; Jenkins, D. J.; Fleming, J. A.; McCrea, C. E.; Pu, S.; Couture, P.; Connelly, P. W.; Jones, P. J.	2016	Effects of canola and high-oleic-acid canola oils on abdominal fat mass in individuals with central obesity
264	Liu, Y.; Chen, H.; Mu, D.; Fan, J.; Song, J.; Zhong, Y.; Li, D.; Xia, M.	2016	Circulating Retinoic Acid Levels and the Development of Metabolic Syndrome
265	Lofgren, I. E.; Herron, K. L.; West, K. L.; Zern, T. L.; Brownbill, R. A.; Ilich, J. Z.; Koo, S. I.; Fernandez, M. L.	2005	Weight loss favorably modifies anthropometrics and reverses the metabolic syndrome in premenopausal women
266	López-Molina, R.; Parra-Cabrera, S.; López-Ridaura, R.; González-Villalpando, M. E.; Ferrannini, E.; González-Villalpando, C.	2013	Sweetened beverages intake, hyperuricemia and metabolic syndrome: the Mexico City Diabetes Study

267	Lu, M. Y.; Cheng, H. Y.; Lai, J. C.; Chen, S. J.	2022	The Relationship between Habitual Coffee Drinking and the Prevalence of Metabolic Syndrome in Taiwanese Adults: Evidence from the Taiwan Biobank Database
268	Luan, D.; Wang, D.; Campos, H.; Baylin, A.	2020	Red meat consumption and metabolic syndrome in the Costa Rica Heart Study
269	Lustig, R. H.; Mulligan, K.; Noworolski, S. M.; Tai, V. W.; Wen, M. J.; Erkin-Cakmak, A.; Gugliucci, A.; Schwarz, J. M.	2016	Isocaloric fructose restriction and metabolic improvement in children with obesity and metabolic syndrome
270	Lutsey, P. L.; Steffen, L. M.; Stevens, J.	2008	Dietary intake and the development of the metabolic syndrome: the Atherosclerosis Risk in Communities study
271	Lv, L.; Yao, Y.; Wang, L.	2011	Dietary glycaemic load and intakes of carbohydrates, fats and proteins in 1040 hospitalised adult Chinese subjects
272	Ma, Y.; Olendzki, B. C.; Wang, J.; Persuitte, G. M.; Li, W.; Fang, H.; Merriam, P. A.; Wedick, N. M.; Ockene, I. S.; Culver, A. L.; Schneider, K. L.; Olendzki, G. F.; Carmody, J.; Ge, T.; Zhang, Z.; Pagoto, S. L.	2015	Single-component versus multicomponent dietary goals for the metabolic syndrome: a randomized trial
273	Madero, M.; Arriaga, J. C.; Jalal, D.; Rivard, C.; McFann, K.; Pérez-Méndez, O.; Vázquez, A.; Ruiz, A.; Lanaspa, M. A.; Jimenez, C. R.; Johnson, R. J.; Lozada, L. G.	2011	The effect of two energy-restricted diets, a low-fructose diet versus a moderate natural fructose diet, on weight loss and metabolic syndrome parameters: a randomized controlled trial
274	Magalhães, Eids; de Oliveira, B. R.; Rudakoff, L. C. S.; de Carvalho, V. A.; Viola, Pcaf; Arruda, S. P. M.; de Carvalho, C. A.; Coelho, Ccnds; Bragança, Mlbm; Bettiol, H.; Barbieri, M. A.; Cardoso, V. C.; Dos Santos, A. M.; Levy, R. B.; da Silva, A. A. M.	2022	Sex-Dependent Effects of the Intake of NOVA Classified Ultra-Processed Foods on Syndrome Metabolic Components in Brazilian Adults

275	Mahdavi, R.; Namazi, N.; Alizadeh, M.; Farajnia, S.	2015	Effects of Nigella sativa oil with a low-calorie diet on cardiometabolic risk factors in obese women: a randomized controlled clinical trial
276	Maki, K. C.; Fulgoni, V. L., 3rd; Keast, D. R.; Rains, T. M.; Park, K. M.; Rubin, M. R.	2012	Vitamin D intake and status are associated with lower prevalence of metabolic syndrome in U.S. adults: National Health and Nutrition Examination Surveys 2003-2006
277	Martinez-Perez, C.; San-Cristobal, R.; Guallar-Castillon, P.; Martínez-González, MÁ; Salas-Salvadó, J.; Corella, D.; Castañer, O.; Martinez, J. A.; Alonso-Gómez Á, M.; Wärnberg, J.; Vioque, J.; Romaguera, D.; López-Miranda, J.; Estruch, R.; Tinahones, F. J.; Lapetra, J.; Serra-Majem, L.; Bueno-Cavanillas, A.; Tur, J. A.; Sánchez, V. M.; Pintó, X.; Gaforio, J. J.; Matía-Martín, P.; Vidal, J.; Vázquez, C.; Ros, E.; Bes-Rastrollo, M.; Babio, N.; Sorlí, J. V.; Lassale, C.; Pérez-Sanz, B.; Vaquero-Luna, J.; Bazán, M. J. A.; Barceló-Iglesias, M. C.; Konieczna, J.; Ríos, A. G.; Bernal-López, M. R.; Santos-Lozano, J. M.; Toledo, E.; Becerra-Tomás, N.; Portoles, O.; Zomeño, M. D.; Abete, I.; Moreno-Rodriguez, A.; Lecea-Juarez, O.; Nishi, S. K.; Muñoz-Martínez, J.; Ordovás, J. M.; Daimiel, L.	2021	Use of Different Food Classification Systems to Assess the Association between Ultra-Processed Food Consumption and Cardiometabolic Health in an Elderly Population with Metabolic Syndrome (PREDIMED-Plus Cohort)
278	Mathews, A. T.; Famodu, O. A.; Olfert, M. D.; Murray, P. J.; Cuff, C. F.; Downes, M. T.; Haughey, N. J.; Colby, S. E.; Chantler,	2017	Efficacy of nutritional interventions to lower circulating ceramides in young adults: FRUVEDomic pilot study

	P. D.; Olfert, I. M.; McFadden, J. W.		
279	Matsumoto, M.; Suganuma, H.; Shimizu, S.; Hayashi, H.; Sawada, K.; Tokuda, I.; Ihara, K.; Nakaji, S.	2020	Skin Carotenoid Level as an Alternative Marker of Serum Total Carotenoid Concentration and Vegetable Intake Correlates with Biomarkers of Circulatory Diseases and Metabolic Syndrome
280	Matsuo, T.; So, R.; Shimojo, N.; Tanaka, K.	2015	Effect of aerobic exercise training followed by a low-calorie diet on metabolic syndrome risk factors in men
281	Matsuura, H.; Mure, K.; Nishio, N.; Kitano, N.; Nagai, N.; Takeshita, T.	2012	Relationship between coffee consumption and prevalence of metabolic syndrome among Japanese civil servants
282	Mattei, J.; Bhupathiraju, S.; Tucker, K. L.	2013	Higher adherence to a diet score based on American Heart Association recommendations is associated with lower odds of allostatic load and metabolic syndrome in Puerto Rican adults
283	Mattei, J.; Malik, V.; Hu, F. B.; Campos, H.	2012	Substituting homemade fruit juice for sugar-sweetened beverages is associated with lower odds of metabolic syndrome among Hispanic adults
284	Mattei, J.; Noel, S. E.; Tucker, K. L.	2011	A meat, processed meat, and French fries dietary pattern is associated with high allostatic load in Puerto Rican older adults
285	Mba, C. M.; Koulman, A.; Forouhi, N. G.; Imamura, F.; Assah, F.; Mbanya, J. C.; Wareham, N. J.	2021	Associations of Serum Folate and Holotranscobalamin with Cardiometabolic Risk Factors in Rural and Urban Cameroon
286	McKeown, N. M.; Meigs, J. B.; Liu, S.; Saltzman, E.; Wilson, P. W.; Jacques, P. F.	2004	Carbohydrate nutrition, insulin resistance, and the prevalence of the metabolic syndrome in the Framingham Offspring Cohort
287	Melero, V.; Arnoriaga, M.; Barabash, A.; Valerio, J.; Del Valle, L.; Martin O'Connor, R.; de Miguel, M. P.; Diaz, J. A.; Familiar, C.; Moraga, I.; Duran, A.; Cuesta, M.; Torrejon, M. J.; Martinez-Novillo, M.; Moreno, M.; Romera, G.; Runkle, I.; Pazos, M.; Rubio, M. A.; Matia-Martín, P.; Calle-Pascual, A. L.	2023	An Early Mediterranean-Based Nutritional Intervention during Pregnancy Reduces Metabolic Syndrome and Glucose Dysregulation Rates at 3 Years Postpartum

288	Mena-Sánchez, G.; Babio, N.; Martínez-González, MÁ; Corella, D.; Schröder, H.; Vioque, J.; Romaguera, D.; Martínez, J. A.; Lopez-Miranda, J.; Estruch, R.; Wärnberg, J.; Bueno-Cavanillas, A.; Serra-Majem, L.; Tur, J. A.; Arós, F.; Tinahones, F. J.; Sánchez, V. M.; Lapetra, J.; Pintó, X.; Vidal, J.; Vázquez, C.; Ordovás, J. M.; Delgado-Rodriguez, M.; Matía-Martín, P.; Basora, J.; Buil-Cosiales, P.; Fernandez-Carrion, R.; Fitó, M.; Salas-Salvadó, J.	2018	Fermented dairy products, diet quality, and cardio-metabolic profile of a Mediterranean cohort at high cardiovascular risk
289	Micek, A.; Grosso, G.; Polak, M.; Kozakiewicz, K.; Tykarski, A.; Puch Walczak, A.; Drygas, W.; Kwaśniewska, M.; Pająk, A.	2018	Association between tea and coffee consumption and prevalence of metabolic syndrome in Poland - results from the WOBASZ II study (2013-2014)
290	Mielgo-Ayuso, J.; Barrenechea, L.; Alcorta, P.; Larrarte, E.; Margareto, J.; Labayen, I.	2014	Effects of dietary supplementation with epigallocatechin-3-gallate on weight loss, energy homeostasis, cardiometabolic risk factors and liver function in obese women: randomised, double-blind, placebo-controlled clinical trial
291	Millar, C. L.; Duclos, Q.; Garcia, C.; Norris, G. H.; Lemos, B. S.; DiMarco, D. M.; Fernandez, M. L.; Blesso, C. N.	2018	Effects of Freeze-Dried Grape Powder on High-Density Lipoprotein Function in Adults with Metabolic Syndrome: A Randomized Controlled Pilot Study
292	Miller, M.; Sorkin, J. D.; Mastella, L.; Sutherland, A.; Rhyne, J.; Donnelly, P.; Simpson, K.; Goldberg, A. P.	2016	Poly is more effective than monounsaturated fat for dietary management in the metabolic syndrome: The muffin study
293	Mirenayat, F. S.; Hajhashemy, Z.; Siavash, M.; Saneei, P.	2023	Effects of sumac supplementation on metabolic markers in adults with metabolic syndrome: a triple-blinded randomized placebo-controlled cross-over clinical trial

294	Mirmiran, P.; Aghayan, M.; Bakhshi, B.; Hosseinpour-Niazi, S.; Azizi, F.	2021	Socioeconomic status and lifestyle factors modifies the association between snack foods intake and incidence of metabolic syndrome
295	Mirmiran, P.; Bahadoran, Z.; Delshad, H.; Azizi, F.	2014	Effects of energy-dense nutrient-poor snacks on the incidence of metabolic syndrome: a prospective approach in Tehran Lipid and Glucose Study
296	Mirmiran, P.; Bakhshi, B.; Hosseinpour-Niazi, S.; Sarbazi, N.; Hejazi, J.; Azizi, F.	2020	Does the association between patterns of fruit and vegetables and metabolic syndrome incidence vary according to lifestyle factors and socioeconomic status?
297	Mirmiran, P.; Gaeini, Z.; Feizy, Z.; Azizi, F.	2023	Dietary fatty acid patterns and risk of metabolic syndrome: Tehran lipid and glucose study
298	Mirmiran, P.; Hosseinpour-Niazi, S.; Naderi, Z.; Bahadoran, Z.; Sadeghi, M.; Azizi, F.	2012	Association between interaction and ratio of ω-3 and ω-6 polyunsaturated fatty acid and the metabolic syndrome in adults
299	Moazzen, H.; Alizadeh, M.	2017	Effects of Pomegranate Juice on Cardiovascular Risk Factors in Patients with Metabolic Syndrome: a Double-Blinded, Randomized Crossover Controlled Trial
300	Moe Å, M.; Ytterstad, E.; Hopstock, L. A.; Løvsletten, O.; Carlsen, M. H.; Sørbye, S. H.	2024	Associations and predictive power of dietary patterns on metabolic syndrome and its components
301	Mohammadi, E.; Rafraf, M.; Farzadi, L.; Asghari-Jafarabadi, M.; Sabour, S.	2012	Effects of omega-3 fatty acids supplementation on serum adiponectin levels and some metabolic risk factors in women with polycystic ovary syndrome
302	Mohammadi-Sartang, M.; Bellissimo, N.; Totosy de Zepetnek, J. O.; Brett, N. R.; Mazloomi, S. M.; Fararouie, M.; Bedeltavana, A.; Famouri, M.; Mazloom, Z.	2018	The effect of daily fortified yogurt consumption on weight loss in adults with metabolic syndrome: A 10-week randomized controlled trial
303	Mohammadifard, N.; Haghighatdoost, F.; Mansourian, M.; Hassannejhad, R.; Sadeghi, M.; Roohafza, H.; Sajjadi, F.; Maghroun, M.; Alikhasi, H.; Sarrafzadegan, N.	2019	Long-term association of nut consumption and cardiometabolic risk factors

304	Moon, S.	2017	Unhealthy Lifestyle Behaviors in Korean People with Metabolic Syndrome
305	Mora-Cubillos, X.; Tulipani, S.; Garcia-Aloy, M.; Bulló, M.; Tinahones, F. J.; Andres-Lacueva, C.	2015	Plasma metabolomic biomarkers of mixed nuts exposure inversely correlate with severity of metabolic syndrome
306	Moreno Franco, B.; León Latre, M.; Andrés Esteban, E. M.; Ordovás, J. M.; Casasnovas, J. A.; Peñalvo, J. L.	2014	Soluble and insoluble dietary fibre intake and risk factors for metabolic syndrome and cardiovascular disease in middle-aged adults: the AWHS cohort
307	Morovati, A.; Pourghassem Gargari, B.; Sarbakhsh, P.	2019	Effects of cumin (Cuminum cyminum L.) essential oil supplementation on metabolic syndrome components: A randomized, triple-blind, placebo-controlled clinical trial
308	Morshedzadeh, N.; Rahimlou, M.; Shahrokh, S.; Karimi, S.; Mirmiran, P.; Zali, M. R.	2021	The effects of flaxseed supplementation on metabolic syndrome parameters, insulin resistance and inflammation in ulcerative colitis patients: An open-labeled randomized controlled trial
309	Mulero, J.; Bernabé, J.; Cerdá, B.; García-Viguera, C.; Moreno, D. A.; Albaladejo, M. D.; Avilés, F.; Parra, S.; Abellán, J.; Zafrilla, P.	2012	Variations on cardiovascular risk factors in metabolic syndrome after consume of a citrus-based juice
310	Murata, G. H.; Shah, J. H.; Duckworth, W. C.; Wendel, C. S.; Mohler, M. J.; Hoffman, R. M.	2004	Food frequency questionnaire results correlate with metabolic control in insulin-treated veterans with type 2 diabetes: the Diabetes Outcomes in Veterans Study
311	Mure, K.; Maeda, S.; Mukoubayashi, C.; Mugitani, K.; Iwane, M.; Kinoshita, F.; Mohara, O.; Takeshita, T.	2013	Habitual coffee consumption inversely associated with metabolic syndrome-related biomarkers involving adiponectin
312	Mutungi, G.; Ratliff, J.; Puglisi, M.; Torres-Gonzalez, M.; Vaishnav, U.; Leite, J. O.; Quann, E.; Volek, J. S.; Fernandez, M. L.	2008	Dietary cholesterol from eggs increases plasma HDL cholesterol in overweight men consuming a carbohydrate-restricted diet
313	Muzio, F.; Mondazzi, L.; Harris, W. S.; Sommariva, D.; Branchi, A.	2007	Effects of moderate variations in the macronutrient content of the diet on cardiovascular disease risk factors in obese patients with the metabolic syndrome

314	Nakandakare, E. R.; Charf, A. M.; Santos, F. C.; Nunes, V. S.; Ortega, K.; Lottenberg, A. M.; Mion, D., Jr.; Nakano, T.; Nakajima, K.; D'Amico, E. A.; Catanozi, S.; Passarelli, M.; Quintão, E. C.	2008	Dietary salt restriction increases plasma lipoprotein and inflammatory marker concentrations in hypertensive patients
315	Nasreddine, L.; Tamim, H.; Itani, L.; Nasrallah, M. P.; Isma'eel, H.; Nakhoul, N. F.; Abou-Rizk, J.; Naja, F.	2018	A minimally processed dietary pattern is associated with lower odds of metabolic syndrome among Lebanese adults
316	Navaei, N.; Pourafshar, S.; Akhavan, N. S.; Litwin, N. S.; Foley, E. M.; George, K. S.; Hartley, S. C.; Elam, M. L.; Rao, S.; Arjmandi, B. H.; Johnson, S. A.	2019	Influence of daily fresh pear consumption on biomarkers of cardiometabolic health in middle-aged/older adults with metabolic syndrome: a randomized controlled trial
317	Negi, P. C.; Sharma, C. K.; Nihjawan, R.; Sharma, R.; Asotra, S.	2022	Role of omega 3 and omega 6 poly unsaturated fatty acids (PUFA) and vitamin D deficiency as risk determinants of metabolic syndrome in obesity: Worksite based case-control observational study
318	Nerurkar, P. V.; Gandhi, K.; Chen, J. J.	2021	Correlations between Coffee Consumption and Metabolic Phenotypes, Plasma Folate, and Vitamin B12: NHANES 2003 to 2006
319	Neuhouser, M. L.; Howard, B.; Lu, J.; Tinker, L. F.; Van Horn, L.; Caan, B.; Rohan, T.; Stefanick, M. L.; Thomson, C. A.	2012	A low-fat dietary pattern and risk of metabolic syndrome in postmenopausal women: the Women's Health Initiative
320	Ngo Njembe, M. T.; Pachikian, B.; Lobysheva, I.; Van Overstraeten, N.; Dejonghe, L.; Verstraelen, E.; Buchet, M.; Rasse, C.; Gardin, C.; Mignolet, E.; Balligand, J. L.; Larondelle, Y.	2021	A Three-Month Consumption of Eggs Enriched with ω-3, ω-5 and ω-7 Polyunsaturated Fatty Acids Significantly Decreases the Waist Circumference of Subjects at Risk of Developing Metabolic Syndrome: A Double-Blind Randomized Controlled Trial
321	Nguyen, H. D.; Oh, H.; Kim, M. S.	2022	Higher intakes of fruits, vegetables, and multiple individual nutrients is associated with a lower risk of metabolic syndrome among adults with comorbidities

322	Nikniaz, L.; Abbasalizad Farhangi, M.; Tabrizi, J. S.; Nikniaz, Z.	2019	Association of major dietary patterns and different metabolic phenotypes: a population-based study of northwestern Iran
323	Nikniaz, L.; Mahmudiono, T.; Jasim, S. A.; Vajdi, M.; Thangavelu, L.; Farhangi, M. A.	2022	Nutrient pattern analysis of mineral based, simple sugar based, and fat based diets and risk of metabolic syndrome: a comparative nutrient panel
324	Noel, S. E.; Newby, P. K.; Ordovas, J. M.; Tucker, K. L.	2009	A traditional rice and beans pattern is associated with metabolic syndrome in Puerto Rican older adults
325	Noori, N.; Nafar, M.; Poorrezagholi, F.; Ahmadpoor, P.; Samadian, F.; Firouzan, A.; Einollahi, B.	2010	Dietary intakes of fiber and magnesium and incidence of metabolic syndrome in first year after renal transplantation
326	O'Neil, C. E.; Fulgoni, V. L., 3rd; Nicklas, T. A.	2011	Candy consumption was not associated with body weight measures, risk factors for cardiovascular disease, or metabolic syndrome in US adults: NHANES 1999-2004
327	O'Neil, C. E.; Fulgoni, V. L., 3rd; Nicklas, T. A.	2015	Tree Nut consumption is associated with better adiposity measures and cardiovascular and metabolic syndrome health risk factors in U.S. Adults: NHANES 2005-2010
328	O'Neil, C. E.; Keast, D. R.; Nicklas, T. A.; Fulgoni, V. L., 3rd	2011	Nut consumption is associated with decreased health risk factors for cardiovascular disease and metabolic syndrome in U.S. adults: NHANES 1999-2004
329	O'Neil, C. E.; Keast, D. R.; Nicklas, T. A.; Fulgoni, V. L., 3rd	2012	Out-of-hand nut consumption is associated with improved nutrient intake and health risk markers in US children and adults: National Health and Nutrition Examination Survey 1999-2004
330	O'Neil, C. E.; Nicklas, T. A.; Rampersaud, G. C.; Fulgoni, V. L., 3rd	2012	100% orange juice consumption is associated with better diet quality, improved nutrient adequacy, decreased risk for obesity, and improved biomarkers of health in adults: National Health and Nutrition Examination Survey, 2003-2006
331	Oh, S. W.; Han, K. H.; Han, S. Y.; Koo, H. S.; Kim, S.; Chin, H. J.	2015	Association of Sodium Excretion With Metabolic Syndrome, Insulin Resistance, and Body Fat
332	Ohta, Y.; Tsuchihashi, T.; Arakawa, K.; Onaka, U.; Ueno, M.	2007	Prevalence and lifestyle characteristics of hypertensive patients with metabolic syndrome followed at an outpatient clinic in fukuoka, Japan
333	Oncina-Cánovas, A.; Vioque, J.; González-Palacios, S.; Martínez-González, MÁ; Salas-	2022	Pro-vegetarian food patterns and cardiometabolic risk in the PREDIMED-Plus study: a cross-sectional baseline analysis

	Salvadó, J.; Corella, D.; Zomeño, D.; Martínez, J. A.; Alonso-Gómez Á, M.; Wärnberg, J.; Romaguera, D.; López-Miranda, J.; Estruch, R.; Bernal-Lopez, R. M.; Lapetra, J.; Serra-Majem, J. L.; Bueno-Cavanillas, A.; Tur, J. A.; Martín-Sánchez, V.; Pintó, X.; Delgado-Rodríguez, M.; Matía-Martín, P.; Vidal, J.; Vázquez, C.; Daimiel, L.; Ros, E.; Toledo, E.; Babio, N.; Sorli, J. V.; Schröder, H.; Zulet, M. A.; Sorto-Sánchez, C.; Barón-López, F. J.; Compañ-Gabucio, L.; Morey, M.; García-Ríos, A.; Casas, R.; Gómez-Pérez, A. M.; Santos-Lozano, J. M.; Vázquez-Ruiz, Z.; Nishi, S. K.; Asensio, E. M.; Soldevila, N.; Abete, I.; Goicolea-Güemez, L.; Buil-Cosiales, P.; García-Gavilán, J. F.; Canals, E.; Torres-Collado, L.; García-de-la-Hera, M.		
334	Otsuka, R.; Imai, T.; Kato, Y.; Ando, F.; Shimokata, H.	2010	Relationship between number of metabolic syndrome components and dietary factors in middle-aged and elderly Japanese subjects
335	Pal, S.; Khossousi, A.; Binns, C.; Dhaliwal, S.; Ellis, V.	2011	The effect of a fibre supplement compared to a healthy diet on body composition, lipids, glucose, insulin and other metabolic syndrome risk factors in overweight and obese individuals
336	Pal, S.; Khossousi, A.; Binns, C.; Dhaliwal, S.; Radavelli-Bagatini, S.	2012	The effects of 12-week psyllium fibre supplementation or healthy diet on blood pressure and arterial stiffness in overweight and obese individuals
337	Palmeiro-Silva, Y. K.; Aravena, R. I.; Ossio, L.; Parro Fluxa, J.	2020	Effects of Daily Consumption of an Aqueous Dispersion of Free-Phytosterols Nanoparticles on Individuals with Metabolic Syndrome: A Randomised, Double-Blind, Placebo-Controlled Clinical Trial

338	Pan, A.; Franco, O. H.; Ye, J.; Demark-Wahnefried, W.; Ye, X.; Yu, Z.; Li, H.; Lin, X.	2008	Soy protein intake has sex-specific effects on the risk of metabolic syndrome in middle-aged and elderly Chinese
339	Pan, F.; Wang, Z.; Wang, H.; Zhang, J.; Su, C.; Jia, X.; Du, W.; Jiang, H.; Li, W.; Wang, L.; Hao, L.; Zhang, B.; Ding, G.	2023	Association between Ultra-Processed Food Consumption and Metabolic Syndrome among Adults in China-Results from the China Health and Nutrition Survey
340	Pang, S.; Song, P.; Sun, X.; Qi, W.; Yang, C.; Song, G.; Wang, Y.; Zhang, J.	2021	Dietary fructose and risk of metabolic syndrome in Chinese residents aged 45 and above: results from the China National Nutrition and Health Survey
341	Pang, T.; Alman, A. C.; Gray, H. L.; Basu, A.; Shi, L.; Snell-Bergeon, J. K.	2021	Empirical dietary inflammatory pattern and metabolic syndrome: prospective association in participants with and without type 1 diabetes mellitus in the coronary artery calcification in type 1 diabetes (CACTI) study
342	Paniagua, J. A.; Pérez-Martinez, P.; Gjelstad, I. M.; Tierney, A. C.; Delgado-Lista, J.; Defoort, C.; Blaak, E. E.; Risérus, U.; Drevon, C. A.; Kiec-Wilk, B.; Lovegrove, J. A.; Roche, H. M.; López-Miranda, J.	2011	A low-fat high-carbohydrate diet supplemented with long-chain n-3 PUFA reduces the risk of the metabolic syndrome
343	Papaioannou, K. G.; Kadi, F.; Nilsson, A.	2022	Benefits of Fruit and Vegetable Consumption on Prevalence of Metabolic Syndrome Are Independent of Physical Activity Behaviors in Older Adults
344	Park, H.; Kityo, A.; Kim, Y.; Lee, S. A.	2021	Macronutrient Intake in Adults Diagnosed with Metabolic Syndrome: Using the Health Examinee (HEXA) Cohort
345	Park, H.; Shin, Y.; Kwon, O.; Kim, Y.	2018	Association of Sensory Liking for Fat with Dietary Intake and Metabolic Syndrome in Korean Adults
346	Park, S.; Ahn, J.; Lee, B. K.	2016	Very-low-fat diets may be associated with increased risk of metabolic syndrome in the adult population
347	Pašalić, D.; Dodig, S.; Corović, N.; Pizent, A.; Jurasović, J.; Pavlović, M.	2011	High prevalence of metabolic syndrome in an elderly Croatian population - a multicentre study
348	Patel, L.; Alicandro, G.; La Vecchia, C.	2018	Low-Calorie Beverage Consumption, Diet Quality and Cardiometabolic Risk Factors in British Adults

349	Pathak, K.; Zhao, Y.; Calton, E. K.; James, A. P.; Newsholme, P.; Sherriff, J.; Soares, M. J.	2024	The impact of leucine supplementation on body composition and glucose tolerance following energy restriction: an 8-week RCT in adults at risk of the metabolic syndrome
350	Pedersen, M. H.; Mølgaard, C.; Hellgren, L. I.; Lauritzen, L.	2010	Effects of fish oil supplementation on markers of the metabolic syndrome
351	Pereira, M. A.; Fulgoni, V. L., 3rd	2010	Consumption of 100% fruit juice and risk of obesity and metabolic syndrome: findings from the national health and nutrition examination survey 1999-2004
352	Pham, T. M.; Ekwaru, J. P.; Setayeshgar, S.; Veugelers, P. J.	2015	The Effect of Changing Serum 25-Hydroxyvitamin D Concentrations on Metabolic Syndrome: A Longitudinal Analysis of Participants of a Preventive Health Program
353	Phillips, N. E.; Mareschal, J.; Schwab, N.; Manoogian, E. N. C.; Borloz, S.; Ostinelli, G.; Gauthier-Jaques, A.; Umwali, S.; Gonzalez Rodriguez, E.; Aeberli, D.; Hans, D.; Panda, S.; Rodondi, N.; Naef, F.; Collet, T. H.	2021	The Effects of Time-Restricted Eating versus Standard Dietary Advice on Weight, Metabolic Health and the Consumption of Processed Food: A Pragmatic Randomised Controlled Trial in Community-Based Adults
354	Pieters, M.; Oosthuizen, W.; Jerling, J. C.; Loots, D. T.; Mukuddem-Petersen, J.; Hanekom, S. M.	2005	Clustering of haemostatic variables and the effect of high cashew and walnut diets on these variables in metabolic syndrome patients
355	Pimenta, A. M.; Bes-Rastrollo, M.; Gea, A.; Sayón-Orea, C.; Zazpe, I.; Lopez-Iracheta, R.; Martinez-Gonzalez, M. A.	2016	Snacking between main meals is associated with a higher risk of metabolic syndrome in a Mediterranean cohort: the SUN Project (Seguimiento Universidad de Navarra)
356	Pimenta, A. M.; Toledo, E.; Rodriguez-Diez, M. C.; Gea, A.; Lopez-Iracheta, R.; Shivappa, N.; Hébert, J. R.; Martinez-Gonzalez, M. A.	2015	Dietary indexes, food patterns and incidence of metabolic syndrome in a Mediterranean cohort: The SUN project
357	Plat, J.; Brufau, G.; Dallinga-Thie, G. M.; Dasselaar, M.; Mensink, R. P.	2009	A plant stanol yogurt drink alone or combined with a low-dose statin lowers serum triacylglycerol and non-HDL cholesterol in metabolic syndrome patients

358	Popiolek-Kalisz, J.	2023	The Relationship between Dietary Flavonols Intake and Metabolic Syndrome in Polish Adults
359	Poppitt, S. D.; Keogh, G. F.; Prentice, A. M.; Williams, D. E.; Sonnemans, H. M.; Valk, E. E.; Robinson, E.; Wareham, N. J.	2002	Long-term effects of ad libitum low-fat, high-carbohydrate diets on body weight and serum lipids in overweight subjects with metabolic syndrome
360	Ramírez-López, G.; Flores-Aldana, M.; Salmerón, J.	2019	Associations between dietary patterns and metabolic syndrome in adolescents
361	Rasaei, N.; Hosseininasab, D.; Shiraseb, F.; Gholami, F.; Noori, S.; Ghaffarian-Ensaf, R.; Daneshzad, E.; Clark, C. C. T.; Mirzaei, K.	2022	The Association between Healthy Beverage Index and Healthy and Unhealthy Obesity Phenotypes among Obese Women: A Cross-Sectional Study
362	Raziani, F.; Tholstrup, T.; Kristensen, M. D.; Svanegaard, M. L.; Ritz, C.; Astrup, A.; Raben, A.	2016	High intake of regular-fat cheese compared with reduced-fat cheese does not affect LDL cholesterol or risk markers of the metabolic syndrome: a randomized controlled trial
363	Relja, A.; Miljković, A.; Gelemanović, A.; Bošković, M.; Hayward, C.; Polašek, O.; Kolčić, I.	2017	Nut Consumption and Cardiovascular Risk Factors: A Cross-Sectional Study in a Mediterranean Population
364	Riseberg, E.; Chui, K.; James, K. A.; Melamed, R.; Alderete, T. L.; Corlin, L.	2022	A Longitudinal Study of Exposure to Manganese and Incidence of Metabolic Syndrome
365	Riseberg, E.; Lopez-Cepero, A.; Mangano, K. M.; Tucker, K. L.; Mattei, J.	2022	Specific Dietary Protein Sources Are Associated with Cardiometabolic Risk Factors in the Boston Puerto Rican Health Study
366	Rodrigues, S. L.; Baldo, M. P.; de Sá Cunha, R.; Andreão, R. V.; Del Carmen Bisi Molina, M.; Gonçalves, C. P.; Dantas, E. M.; Mill, J. G.	2009	Salt excretion in normotensive individuals with metabolic syndrome: a population-based study
367	Rodríguez-Moran, M.; Guerrero-Romero, F.	2014	Oral magnesium supplementation improves the metabolic profile of metabolically obese, normal-weight individuals: a randomized double-blind placebo-controlled trial

368	Root, M. M.; Dawson, H. R.	2013	DASH-like diets high in protein or monounsaturated fats improve metabolic syndrome and calculated vascular risk
369	Rosell, M.; De Faire, U.; Hellénius, M. L.	2003	Low prevalence of the metabolic syndrome in wine drinkers--is it the alcohol beverage or the lifestyle?
370	Ruidavets, J. B.; Bongard, V.; Dallongeville, J.; Arveiler, D.; Ducimetière, P.; Perret, B.; Simon, C.; Amouyel, P.; Ferrières, J.	2007	High consumptions of grain, fish, dairy products and combinations of these are associated with a low prevalence of metabolic syndrome
371	Ruscica, M.; Pavanello, C.; Gandini, S.; Gomaraschi, M.; Vitali, C.; Macchi, C.; Morlotti, B.; Aiello, G.; Bosisio, R.; Calabresi, L.; Arnoldi, A.; Sirtori, C. R.; Magni, P.	2018	Effect of soy on metabolic syndrome and cardiovascular risk factors: a randomized controlled trial
372	Sabir, A. A.; Bilbis, L. S.; Saidu, Y.; Jimoh, A.; Iwuala, S. O.; Isezuo, S. A.; Kaoje, A. U.; Abubakar, S. A.	2016	Oxidative stress among subjects with metabolic syndrome in Sokoto, North-Western Nigeria
373	Safavi, M.; Farajian, S.; Kelishadi, R.; Mirlohi, M.; Hashemipour, M.	2013	The effects of synbiotic supplementation on some cardio-metabolic risk factors in overweight and obese children: a randomized triple-masked controlled trial
374	Salas-Salvadó, J.; Fernández-Ballart, J.; Ros, E.; Martínez-González, M. A.; Fitó, M.; Estruch, R.; Corella, D.; Fiol, M.; Gómez-Gracia, E.; Arós, F.; Flores, G.; Lapetra, J.; Lamuela-Raventós, R.; Ruiz-Gutiérrez, V.; Bulló, M.; Basora, J.; Covas, M. I.	2008	Effect of a Mediterranean diet supplemented with nuts on metabolic syndrome status: one-year results of the PREDIMED randomized trial
375	Salekzamani, S.; Mehralizadeh, H.; Ghezel, A.; Salekzamani, Y.; Jafarabadi, M. A.; Bavil, A. S.; Gargari, B. P.	2016	Effect of high-dose vitamin D supplementation on cardiometabolic risk factors in subjects with metabolic syndrome: a randomized controlled double-blind clinical trial

376	Sanchez-Rodriguez, E.; Lima-Cabello, E.; Biel-Glesson, S.; Fernandez-Navarro, J. R.; Calleja, M. A.; Roca, M.; Espejo-Calvo, J. A.; Gil-Extremera, B.; Soria-Florido, M.; de la Torre, R.; Fito, M.; Covas, M. I.; Alche, J. D.; Martinez de Victoria, E.; Gil, A.; Mesa, M. D.	2018	Effects of Virgin Olive Oils Differing in Their Bioactive Compound Contents on Metabolic Syndrome and Endothelial Functional Risk Biomarkers in Healthy Adults: A Randomized Double-Blind Controlled Trial
377	Saneei, P.; Fallahi, E.; Barak, F.; Ghasemifard, N.; Keshteli, A. H.; Yazdannik, A. R.; Esmaillzadeh, A.	2015	Adherence to the DASH diet and prevalence of the metabolic syndrome among Iranian women
378	Sangouni, A. A.; Alizadeh, M.; Jamalzehi, A.; Parastouei, K.	2021	Effects of garlic powder supplementation on metabolic syndrome components, insulin resistance, fatty liver index, and appetite in subjects with metabolic syndrome: A randomized clinical trial
379	Saraf-Bank, S.; Haghighatdoost, F.; Esmaillzadeh, A.; Larijani, B.; Azadbakht, L.	2017	Adherence to Healthy Eating Index-2010 is inversely associated with metabolic syndrome and its features among Iranian adult women
380	Sayón-Orea, C.; Bes-Rastrollo, M.; Martí, A.; Pimenta, A. M.; Martín-Calvo, N.; Martínez-González, M. A.	2015	Association between yogurt consumption and the risk of metabolic syndrome over 6 years in the SUN study
381	Schioldan, A. G.; Gregersen, S.; Hald, S.; Bjørnshave, A.; Bohl, M.; Hartmann, B.; Holst, J. J.; Stødkilde-Jørgensen, H.; Hermansen, K.	2018	Effects of a diet rich in arabinoxylan and resistant starch compared with a diet rich in refined carbohydrates on postprandial metabolism and features of the metabolic syndrome
382	Sebastian, R. S.; Fanelli Kuczmarski, M. T.; Goldman, J. D.; Moshfegh, A. J.; Zonderman, A. B.; Evans, M. K.	2022	Usual Intake of Flavonoids Is Inversely Associated with Metabolic Syndrome in African American and White Males but Not Females in Baltimore City, Maryland, USA
383	Sekgala, M. D.; McHiza, Z. J.; Parker, W. A.; Monyeki, K. D.	2018	Dietary Fiber Intake and Metabolic Syndrome Risk Factors among Young South African Adults

384	Sekgala, M. D.; Opperman, M.; Mpahleni, B.; McHiza, Z. J.	2022	Association between Macronutrient and Fatty Acid Consumption and Metabolic Syndrome: A South African Taxi Driver Survey
385	Seo, E. H.; Kim, H.; Kwon, O.	2019	Association between Total Sugar Intake and Metabolic Syndrome in Middle-Aged Korean Men and Women
386	Shabrina, A.; Tung, T. H.; Nguyen, N. T. K.; Lee, H. C.; Wu, H. T.; Wang, W.; Huang, S. Y.	2020	-3 PUFA and caloric restriction diet alters lipidomic profiles in obese men with metabolic syndrome: a preliminary open study
387	Shafique, K.; Zafar, M.; Ahmed, Z.; Khan, N. A.; Mughal, M. A.; Imtiaz, F.	2013	Areca nut chewing and metabolic syndrome: evidence of a harmful relationship
388	Sharafedtinov, K. K.; Plotnikova, O. A.; Alexeeva, R. I.; Sentsova, T. B.; Songisepp, E.; Stsepetova, J.; Smidt, I.; Mikelsaar, M.	2013	Hypocaloric diet supplemented with probiotic cheese improves body mass index and blood pressure indices of obese hypertensive patients--a randomized double-blind placebo-controlled pilot study
389	Shearer, G. C.; Pottala, J. V.; Hansen, S. N.; Brandenburg, V.; Harris, W. S.	2012	Effects of prescription niacin and omega-3 fatty acids on lipids and vascular function in metabolic syndrome: a randomized controlled trial
390	Shenoy, S. F.; Poston, W. S.; Reeves, R. S.; Kazaks, A. G.; Holt, R. R.; Keen, C. L.; Chen, H. J.; Haddock, C. K.; Winters, B. L.; Khoo, C. S.; Foreyt, J. P.	2010	Weight loss in individuals with metabolic syndrome given DASH diet counseling when provided a low sodium vegetable juice: a randomized controlled trial
391	Sherafat-Kazemzadeh, R.; Egtesadi, S.; Mirmiran, P.; Gohari, M.; Farahani, S. J.; Esfahani, F. H.; Vafa, M. R.; Hedayati, M.; Azizi, F.	2010	Dietary patterns by reduced rank regression predicting changes in obesity indices in a cohort study: Tehran Lipid and Glucose Study
392	Shi, J.; He, L.; Yu, D.; Ju, L.; Guo, Q.; Piao, W.; Xu, X.; Zhao, L.; Yuan, X.; Cao, Q.; Fang, H.	2022	Prevalence and Correlates of Metabolic Syndrome and Its Components in Chinese Children and Adolescents Aged 7-17: The China National Nutrition and Health Survey of Children and Lactating Mothers from 2016-2017
393	Shin, H. J.; Cho, E.; Lee, H. J.; Fung, T. T.; Rimm, E.; Rosner, B.; Manson, J. E.; Wheelan, K.; Hu, F. B.	2014	Instant noodle intake and dietary patterns are associated with distinct cardiometabolic risk factors in Korea

394	Shin, S.; Kim, S. A.; Ha, J.; Lim, K.	2018	Sugar-Sweetened Beverage Consumption in Relation to Obesity and Metabolic Syndrome among Korean Adults: A Cross-Sectional Study from the 2012⁻2016 Korean National Health and Nutrition Examination Survey (KNHANES)
395	Shin, S.; Lee, H. W.; Kim, C. E.; Lim, J.; Lee, J. K.; Kang, D.	2017	Association between Milk Consumption and Metabolic Syndrome among Korean Adults: Results from the Health Examinees Study
396	Sialvera, T. E.; Pounis, G. D.; Koutelidakis, A. E.; Richter, D. J.; Yfanti, G.; Kapsokefalou, M.; Goumas, G.; Chiotinis, N.; Diamantopoulos, E.; Zampelas, A.	2012	Phytosterols supplementation decreases plasma small and dense LDL levels in metabolic syndrome patients on a westernized type diet
397	Silveira, J. Q.; Dourado, G. K.; Cesar, T. B.	2015	Red-fleshed sweet orange juice improves the risk factors for metabolic syndrome
398	Simão, A. N.; Lozovoy, M. A.; Bahls, L. D.; Morimoto, H. K.; Simão, T. N.; Matsuo, T.; Dichi, I.	2012	Blood pressure decrease with ingestion of a soya product (kinako) or fish oil in women with the metabolic syndrome: role of adiponectin and nitric oxide
399	Simão, A. N.; Lozovoy, M. A.; Simão, T. N.; Dichi, J. B.; Matsuo, T.; Dichi, I.	2010	Nitric oxide enhancement and blood pressure decrease in patients with metabolic syndrome using soy protein or fish oil
400	Simpson, E. J.; Mendis, B.; Macdonald, I. A.	2016	Orange juice consumption and its effect on blood lipid profile and indices of the metabolic syndrome; a randomised, controlled trial in an at-risk population
401	Sivaprakasapillai, B.; Edirisinghe, I.; Randolph, J.; Steinberg, F.; Kappagoda, T.	2009	Effect of grape seed extract on blood pressure in subjects with the metabolic syndrome
402	Skilton, M. R.; Laville, M.; Cust, A. E.; Moulin, P.; Bonnet, F.	2008	The association between dietary macronutrient intake and the prevalence of the metabolic syndrome
403	Skórkowska-Telichowska, K.; Kosińska, J.; Chwojnicka, M.; Tuchendler, D.; Tabin, M.; Tuchendler, R.; Bobak, Ł; Trziszka, T.; Szuba, A.	2016	Positive effects of egg-derived phospholipids in patients with metabolic syndrome
404	Šmíd, V.; Dvořák, K.; Šedivý, P.; Kosek, V.; Leníček, M.;	2022	Effect of Omega-3 Polyunsaturated Fatty Acids on Lipid Metabolism in Patients With Metabolic Syndrome and NAFLD

	Dezortová, M.; Hajšlová, J.; Hájek, M.; Vítek, L.; Bechyňská, K.; Brůha, R.		
405	So, C. H.; Jeong, H. R.; Shim, Y. S.	2017	Association of the urinary sodium to urinary specific gravity ratio with metabolic syndrome in Korean children and adolescents: The Korea National Health and Nutrition Examination Survey 2010-2013
406	Soltani, N.; Farhangi, M. A.; Nikniaz, L.; Mahmoudinezhad, M.	2020	Association between a novel dietary lipophilic index (LI) with metabolic phenotypes in a community-based study in Tabriz- Iran
407	Song, P.; Zhang, X.; Li, Y.; Man, Q.; Jia, S.; Zhang, J.; Ding, G.	2022	MetS Prevalence and Its Association with Dietary Patterns among Chinese Middle-Aged and Elderly Population: Results from a National Cross-Sectional Study
408	Song, S.; Song, Y.	2021	Dietary Fiber and Its Source Are Associated with Cardiovascular Risk Factors in Korean Adults
409	Sorrentino, G.; Crispino, P.; Coppola, D.; De Stefano, G.	2015	Efficacy of lifestyle changes in subjects with non-alcoholic liver steatosis and metabolic syndrome may be improved with an antioxidant nutraceutical: a controlled clinical study
410	Standage-Beier, C. S.; Bakhshi, B.; Parra, O. D.; Soltani, L.; Spegman, D. J.; Molina, P.; Pereira, E.; Landes, L.; Mandarino, L. J.; Kohler, L. N.	2022	Fruit, Vegetable, and Physical Activity Guideline Adherence and Metabolic Syndrome in El Banco por Salud
411	Steemburgo, T.; Dall'Alba, V.; Almeida, J. C.; Zelmanovitz, T.; Gross, J. L.; de Azevedo, M. J.	2009	Intake of soluble fibers has a protective role for the presence of metabolic syndrome in patients with type 2 diabetes
412	Su, H. Y.; Lee, H. C.; Cheng, W. Y.; Huang, S. Y.	2015	A calorie-restriction diet supplemented with fish oil and high-protein powder is associated with reduced severity of metabolic syndrome in obese women
413	Suliga, E.; Ciesla, E.; Lelonek, M.; Piechowska, A.; Gluszek, S.	2022	Lifestyle elements and risk of metabolic syndrome in adults
414	Suliga, E.; Kozieł, D.; Cieśla, E.; Rębak, D.; Głuszek, S.	2017	Coffee consumption and the occurrence and intensity of metabolic syndrome: a cross-sectional study
415	Sumislawski, K.; Widmer, A.; Suro, R. R.; Robles, M. E.; Lillegard,	2023	Consumption of Tree Nuts as Snacks Reduces Metabolic Syndrome Risk in Young Adults: A Randomized Trial

	K.; Olson, D.; Koethe, J. R.; Silver, H. J.		
416	Sun, S. Z.; Anderson, G. H.; Flickinger, B. D.; Williamson-Hughes, P. S.; Empie, M. W.	2011	Fructose and non-fructose sugar intakes in the US population and their associations with indicators of metabolic syndrome
417	Syauqy, A.; Hsu, C. Y.; Rau, H. H.; Chao, J. C.	2018	Association of Dietary Patterns with Components of Metabolic Syndrome and Inflammation among Middle-Aged and Older Adults with Metabolic Syndrome in Taiwan
418	Takagi, T.; Hayashi, R.; Nakai, Y.; Okada, S.; Miyashita, R.; Yamada, M.; Mihara, Y.; Mizushima, K.; Morita, M.; Uchiyama, K.; Naito, Y.; Itoh, Y.	2020	Dietary Intake of Carotenoid-Rich Vegetables Reduces Visceral Adiposity in Obese Japanese men-A Randomized, Double-Blind Trial
419	Takami, H.; Nakamoto, M.; Uemura, H.; Katsuura, S.; Yamaguchi, M.; Hiyoshi, M.; Sawachika, F.; Juta, T.; Arisawa, K.	2013	Inverse correlation between coffee consumption and prevalence of metabolic syndrome: baseline survey of the Japan Multi-Institutional Collaborative Cohort (J-MICC) Study in Tokushima, Japan
420	Tardivo, A. P.; Nahas-Neto, J.; Nahas, E. A.; Maesta, N.; Rodrigues, M. A.; Orsatti, F. L.	2010	Associations between healthy eating patterns and indicators of metabolic risk in postmenopausal women
421	Tardivo, A. P.; Nahas-Neto, J.; Orsatti, C. L.; Dias, F. B.; Poloni, P. F.; Schmitt, E. B.; Nahas, E. A.	2015	Effects of omega-3 on metabolic markers in postmenopausal women with metabolic syndrome
422	te Velde, S. J.; Snijder, M. B.; van Dijk, A. E.; Brug, J.; Koppes, L. L.; van Mechelen, W.; Twisk, J. W.	2011	Dairy intake from adolescence into adulthood is not associated with being overweight and metabolic syndrome in adulthood: the Amsterdam Growth and Health Longitudinal Study
423	Teramoto, T.; Kawamori, R.; Miyazaki, S.; Teramukai, S.	2011	Sodium intake in men and potassium intake in women determine the prevalence of metabolic syndrome in Japanese hypertensive patients: OMEGA Study
424	Thomas, M. S.; Puglisi, M.; Malysheva, O.; Caudill, M. A.; Sholola, M.; Cooperstone, J. L.; Fernandez, M. L.	2022	Eggs Improve Plasma Biomarkers in Patients with Metabolic Syndrome Following a Plant-Based Diet-A Randomized Crossover Study

425	Thorp, A. A.; McNaughton, S. A.; Owen, N.; Dunstan, D. W.	2013	Independent and joint associations of TV viewing time and snack food consumption with the metabolic syndrome and its components; a cross-sectional study in Australian adults
426	Tierney, A. C.; McMonagle, J.; Shaw, D. I.; Gulseth, H. L.; Helal, O.; Saris, W. H.; Paniagua, J. A.; Gołąbek-Leszczyñska, I.; Defoort, C.; Williams, C. M.; Karsltröm, B.; Vessby, B.; Dembinska-Kiec, A.; López-Miranda, J.; Blaak, E. E.; Drevon, C. A.; Gibney, M. J.; Lovegrove, J. A.; Roche, H. M.	2011	Effects of dietary fat modification on insulin sensitivity and on other risk factors of the metabolic syndrome--LIPGENE: a European randomized dietary intervention study
427	Tørris, C.; Molin, M.; Cvancarova, M. S.	2016	Lean fish consumption is associated with lower risk of metabolic syndrome: a Norwegian cross sectional study
428	Trejo-Reyes, R.; Cantoral, A.; Lamadrid-Figueroa, H.; Betanzos-Robledo, L.; Téllez-Rojo, M. M.; Peterson, K. E.; Baylin, A.; Jansen, E. C.	2023	Plasma Fatty Acid Biomarkers of Dairy Consumption Are Associated with Sex-Dependent Effects on Metabolic Syndrome Components in Mexican Adolescents
429	Tremblay, A.; Clinchamps, M.; Pereira, B.; Courteix, D.; Lesourd, B.; Chapier, R.; Obert, P.; Vinet, A.; Walther, G.; Chaplais, E.; Bagheri, R.; Baker, J. S.; Thivel, D.; Drapeau, V.; Dutheil, F.	2020	Dietary Fibres and the Management of Obesity and Metabolic Syndrome: The RESOLVE Study
430	Tresserra-Rimbau, A.; Medina-Remón, A.; Lamuela-Raventós, R. M.; Bulló, M.; Salas-Salvadó, J.; Corella, D.; Fitó, M.; Gea, A.; Gómez-Gracia, E.; Lapetra, J.; Arós, F.; Fiol, M.; Ros, E.; Serra-Majem, L.; Pintó, X.; Muñoz, M. A.; Estruch, R.	2015	Moderate red wine consumption is associated with a lower prevalence of the metabolic syndrome in the PREDIMED population

431	Tsai, C. H.; Chen, E. C.; Tsay, H. S.; Huang, C. J.	2012	Wild bitter gourd improves metabolic syndrome: a preliminary dietary supplementation trial
432	Tsitsimpikou, C.; Tsarouhas, K.; Kioukia-Fougia, N.; Skondra, C.; Fragkiadaki, P.; Papalexis, P.; Stamatopoulos, P.; Kaplanis, I.; Hayes, A. W.; Tsatsakis, A.; Rentoukas, E.	2014	Dietary supplementation with tomato-juice in patients with metabolic syndrome: a suggestion to alleviate detrimental clinical factors
433	Tuomainen, M.; Kärkkäinen, O.; Leppänen, J.; Auriola, S.; Lehtonen, M.; Savolainen, M. J.; Hermansen, K.; Risérus, U.; Åkesson, B.; Thorsdottir, I.; Kolehmainen, M.; Uusitupa, M.; Poutanen, K.; Schwab, U.; Hanhineva, K.	2019	Quantitative assessment of betainized compounds and associations with dietary and metabolic biomarkers in the randomized study of the healthy Nordic diet (SYSDIET)
434	Tureck, C.; Retondario, A.; de Moura Souza, A.; Barboza, B. P.; Bricarello, L. P.; Alves, M. A.; de Vasconcelos, F. A. G.	2023	Omega-3 and omega-6 fatty acids food intake and metabolic syndrome in adolescents 12 to 17 years old: A school-based cross-sectional study
435	Unal, A.; Kocyigit, I.; Sipahioglu, M. H.; Tokgoz, B.; Oymak, O.	2014	The relationship between daily urinary sodium excretion and metabolic syndrome in patients with kidney transplantation
436	Usharani, P.; Merugu, P. L.; Nutalapati, C.	2019	Evaluation of the effects of a standardized aqueous extract of Phyllanthus emblica fruits on endothelial dysfunction, oxidative stress, systemic inflammation and lipid profile in subjects with metabolic syndrome: a randomised, double blind, placebo controlled clinical study
437	Ushula, T. W.; Mamun, A.; Darssan, D.; Wang, W. Y. S.; Williams, G. M.; Whiting, S. J.; Najman, J. M.	2022	Dietary patterns and the risks of metabolic syndrome and insulin resistance among young adults: Evidence from a longitudinal study
438	Usui, T.; Tochiya, M.; Sasaki, Y.; Muranaka, K.; Yamakage, H.; Himeno, A.; Shimatsu, A.; Inaguma, A.; Ueno,	2013	Effects of natural S-equol supplements on overweight or obesity and metabolic syndrome in the Japanese, based on sex and equol status

	T.; Uchiyama, S.; Satoh-Asahara, N.		
439	van Hees, A. M.; Jocken, J. W.; Essers, Y.; Roche, H. M.; Saris, W. H.; Blaak, E. E.	2012	Adipose triglyceride lipase and hormone-sensitive lipase protein expression in subcutaneous adipose tissue is decreased after an isoenergetic low-fat high-complex carbohydrate diet in the metabolic syndrome
440	van Nielen, M.; Feskens, E. J.; Rietman, A.; Siebelink, E.; Mensink, M.	2014	Partly replacing meat protein with soy protein alters insulin resistance and blood lipids in postmenopausal women with abdominal obesity
441	Vanhala, M.; Saltevo, J.; Soininen, P.; Kautiainen, H.; Kangas, A. J.; Ala-Korpela, M.; Mäntyselkä, P.	2012	Serum omega-6 polyunsaturated fatty acids and the metabolic syndrome: a longitudinal population-based cohort study
442	Vázquez, C.; Botella-Carretero, J. I.; Corella, D.; Fiol, M.; Lage, M.; Lurbe, E.; Richart, C.; Fernández-Real, J. M.; Fuentes, F.; Ordóñez, A.; de Cos, A. I.; Salas-Salvadó, J.; Burguera, B.; Estruch, R.; Ros, E.; Pastor, O.; Casanueva, F. F.	2014	White fish reduces cardiovascular risk factors in patients with metabolic syndrome: the WISH-CARE study, a multicenter randomized clinical trial
443	Velasquez-Melendez, G.; Molina, M. D.; Benseñor, I. M.; Cardoso, L. O.; Fonseca, M. J.; Moreira, A. D.; Pereira, T. S.; Barreto, S. M.	2017	Sweetened Soft Drinks Consumption Is Associated with Metabolic Syndrome: Cross-sectional Analysis from the Brazilian Longitudinal Study of Adult Health (ELSA-Brasil)
444	Veldhuis, L.; Koppes, L. L.; Driessen, M. T.; Samoocha, D.; Twisk, J. W.	2010	Effects of dietary fibre intake during adolescence on the components of the metabolic syndrome at the age of 36 years: the Amsterdam Growth and Health Longitudinal Study
445	Ventura, E. E.; Davis, J. N.; Alexander, K. E.; Shaibi, G. Q.; Lee, W.; Byrd-Williams, C. E.; Toledo-Corral, C. M.; Lane, C. J.; Kelly, L. A.; Weigensberg, M. J.; Goran, M. I.	2008	Dietary intake and the metabolic syndrome in overweight Latino children
446	Venturini, D.; Simão, A. N.; Urbano, M. R.; Dichi, I.	2015	Effects of extra virgin olive oil and fish oil on lipid profile and oxidative stress in patients with metabolic syndrome

447	Vernarelli, J. A.; Lambert, J. D.	2013	Tea consumption is inversely associated with weight status and other markers for metabolic syndrome in US adults
448	Verrusio, W.; Andreozzi, P.; Renzi, A.; Musumeci, M.; Gueli, N.; Cacciafesta, M.	2017	Association between serum vitamin D and metabolic syndrome in middle-aged and older adults and role of supplementation therapy with vitamin D
449	Veum, V. L.; Laupsa-Borge, J.; Eng, Ø; Rostrup, E.; Larsen, T. H.; Nordrehaug, J. E.; Nygård, O. K.; Sagen, J. V.; Gudbrandsen, O. A.; Dankel, S. N.; Mellgren, G.	2017	Visceral adiposity and metabolic syndrome after very high-fat and low-fat isocaloric diets: a randomized controlled trial
450	Villaça Chaves, G.; Gonçalves de Souza, G.; Cardoso de Matos, A.; Abrantes Peres, W.; Pereira, S. E.; Saboya, C. J.; D'Almeida, C. A.; Ramalho, A.	2010	Serum retinol and β-carotene levels and risk factors for cardiovascular disease in morbid obesity
451	Villatoro-Santos, C. R.; Ramirez-Zea, M.; Villamor, E.	2022	Urinary sodium, iodine, and volume in relation to metabolic syndrome in Mesoamerican children and their parents
452	Vuksan, V.; Sievenpiper, J. L.; Jovanovski, E.; Jenkins, A. L.; Komishon, A.; Au-Yeung, F.; Zurbau, A.; Ho, H. V. T.; Li, D.; Smircic-Duvnjak, L.	2020	Effect of soluble-viscous dietary fibre on coronary heart disease risk score across 3 population health categories: data from randomized, double-blind, placebo-controlled trials
453	Vulevic, J.; Juric, A.; Tzortzis, G.; Gibson, G. R.	2013	A mixture of trans-galactooligosaccharides reduces markers of metabolic syndrome and modulates the fecal microbiota and immune function of overweight adults
454	Wan, X.; Zhu, F.; Zhuang, P.; Liu, X.; Zhang, L.; Jia, W.; Jiao, J.; Xu, C.; Zhang, Y.	2022	Associations of Hemoglobin Adducts of Acrylamide and Glycidamide with Prevalent Metabolic Syndrome in a Nationwide Population-Based Study
455	Wang, J. W.; Mark, S.; Henderson, M.; O'Loughlin, J.; Tremblay, A.; Wortman, J.; Paradis, G.; Gray-Donald, K.	2013	Adiposity and glucose intolerance exacerbate components of metabolic syndrome in children consuming sugar-sweetened beverages: QUALITY cohort study
456	Wang, S.; Han, Y.; Zhao, H.; Han, X.; Yin, Y.; Wu, J.; Zhang, Y.; Zeng, X.	2022	Association between Coffee Consumption, Caffeine Intake, and Metabolic Syndrome Severity in Patients with Self-Reported

			Rheumatoid Arthritis: National Health and Nutrition Examination Survey 2003-2018
457	Wang, Y.; Dai, Y.; Tian, T.; Zhang, J.; Xie, W.; Pan, D.; Xu, D.; Lu, Y.; Wang, S.; Xia, H.; Sun, G.	2021	The Effects of Dietary Pattern on Metabolic Syndrome in Jiangsu Province of China: Based on a Nutrition and Diet Investigation Project in Jiangsu Province
458	Wang, Y. J.; Chien, K. L.; Hsu, H. C.; Lin, H. J.; Su, T. C.; Chen, M. F.; Lee, Y. T.	2022	Urinary sodium excretion and the risk of CVD: a community-based cohort study in Taiwan
459	Wastyk, H. C.; Perelman, D.; Topf, M.; Fragiadakis, G. K.; Robinson, J. L.; Sonnenburg, J. L.; Gardner, C. D.; Sonnenburg, E. D.	2023	Randomized controlled trial demonstrates response to a probiotic intervention for metabolic syndrome that may correspond to diet
460	Watanabe, T.; Arisawa, K.; Nguyen, T. V.; Ishizu, M.; Katsuura-Kamano, S.; Hishida, A.; Tamura, T.; Kato, Y.; Okada, R.; Ibusuki, R.; Koriyama, C.; Suzuki, S.; Otani, T.; Koyama, T.; Tomida, S.; Kuriki, K.; Takashima, N.; Miyagawa, N.; Wakai, K.; Matsuo, K.	2023	Coffee and metabolic phenotypes: A cross-sectional analysis of the Japan multi-institutional collaborative cohort (J-MICC) study
461	Wei, J.; Zeng, C.; Gong, Q. Y.; Li, X. X.; Lei, G. H.; Yang, T. B.	2015	Associations between Dietary Antioxidant Intake and Metabolic Syndrome
462	Wei, L.; Fan, J.; Dong, R.; Zhang, M.; Jiang, Y.; Zhao, Q.; Zhao, G.; Chen, B.; Li, J.; Liu, S.	2023	The Effect of Dietary Pattern on Metabolic Syndrome in a Suburban Population in Shanghai, China
463	Wennersberg, M. H.; Smedman, A.; Turpeinen, A. M.; Retterstøl, K.; Tengblad, S.; Lipre, E.; Aro, A.; Mutanen, P.; Seljeflot, I.; Basu, S.; Pedersen, J. I.; Mutanen, M.; Vessby, B.	2009	Dairy products and metabolic effects in overweight men and women: results from a 6-mo intervention study

464	Wirfält, E.; Hedblad, B.; Gullberg, B.; Mattisson, I.; Andrén, C.; Rosander, U.; Janzon, L.; Berglund, G.	2001	Food patterns and components of the metabolic syndrome in men and women: a cross-sectional study within the Malmö Diet and Cancer cohort
465	Won, J. C.; Hong, J. W.; Noh, J. H.; Kim, D. J.	2016	Association Between Estimated 24-h Urinary Sodium Excretion and Metabolic Syndrome in Korean Adults: The 2009 to 2011 Korea National Health and Nutrition Examination Survey
466	Wong, T. H. T.; Burlutsky, G.; Gopinath, B.; Flood, V. M.; Mitchell, P.; Louie, J. C. Y.	2022	The longitudinal association between coffee and tea consumption and the risk of metabolic syndrome and its component conditions in an older adult population
467	Wong, T. H. T.; George, E. S.; Abbott, G.; Daly, R. M.; Georgousopoulou, E. N.; Tan, S. Y.	2023	Nut and seed consumption is inversely associated with metabolic syndrome in females but not males: findings from the 2005-2018 NHANES data
468	Wongwiwatthananukit, S.; Sansanayudh, N.; Phetkrajaysang, N.; Krittiyanunt, S.	2013	Effects of vitamin D(2) supplementation on insulin sensitivity and metabolic parameters in metabolic syndrome patients
469	Woo, H. W.; Kim, M. K.; Lee, Y. H.; Shin, D. H.; Shin, M. H.; Choi, B. Y.	2019	Habitual consumption of soy protein and isoflavones and risk of metabolic syndrome in adults ≥ 40 years old: a prospective analysis of the Korean Multi-Rural Communities Cohort Study (MRCohort)
470	Woo, H. W.; Lim, Y. H.; Kim, M. K.; Shin, J.; Lee, Y. H.; Shin, D. H.; Shin, M. H.; Choi, B. Y.	2020	Prospective associations between total, animal, and vegetable calcium intake and metabolic syndrome in adults aged 40 years and older
471	Wu, F.; Zhuang, P.; Zhan, C.; Shen, X.; Jiao, J.; Zhang, Y.	2022	Egg and Dietary Cholesterol Consumption and the Prevalence of Metabolic Syndrome: Findings from a Population-Based Nationwide Cohort
472	Wu, P. W.; Tsai, S.; Lee, C. Y.; Lin, W. T.; Chin, Y. T.; Huang, H. L.; Seal, D. W.; Chen, T.; Lee, C. H.	2021	Contribution of insulin resistance to the relationship between sugar-sweetened beverage intake and a constellation of cardiometabolic abnormalities in adolescents
473	Wu, X.; Unno, T.; Kang, S.; Park, S.	2021	A Korean-Style Balanced Diet Has a Potential Connection with Ruminococcaceae Enterotype and Reduction of Metabolic Syndrome Incidence in Korean Adults
474	Xu, F.; Fan, W.; Wang, W.; Tang, W.; Yang, F.; Zhang, Y.; Cai, J.; Song, L.; Zhang, C.	2019	Effects of omega-3 fatty acids on metabolic syndrome in patients with schizophrenia: a 12-week randomized placebo-controlled trial

475	Yang, H. J.; Song, M. Y.; Kim, M. J.; Park, S.	2020	Associations between metabolic syndrome and urinary Na-to-K ratio and glomerular filtration rate in middle-aged adults regardless of Na and K intakes
476	Yang, J.; Huang, J.; Huang, Z.; Xu, Y.; Li, W.; Zhu, S.; Zhao, Y.; Ye, B.; Liu, L.; Zhu, J.; Xia, M.; Liu, Y.	2023	Cardiometabolic benefits of Lacticaseibacillus paracasei 8700:2: A randomized double-blind placebo-controlled trial
477	Yang, T. Y.; Chou, J. I.; Ueng, K. C.; Chou, M. Y.; Yang, J. J.; Lin-Shiau, S. Y.; Hu, M. E.; Lin, J. K.	2014	Weight reduction effect of Puerh tea in male patients with metabolic syndrome
478	Yang, Y.; Yu, D.; Piao, W.; Huang, K.; Zhao, L.	2022	Association between Habitual Tea Consumption and Metabolic Syndrome and Its Components among Chinese Adults Aged 18~59 Years: Based on China Nutrition and Health Surveillance 2015-2017
479	Yari, Z.; Cheraghpour, M.; Hekmatdoost, A.	2021	Flaxseed and/or hesperidin supplementation in metabolic syndrome: an open-labeled randomized controlled trial
480	Yari, Z.; Rahimlou, M.; Poustchi, H.; Hekmatdoost, A.	2016	Flaxseed Supplementation in Metabolic Syndrome Management: A Pilot Randomized, Open-labeled, Controlled Study
481	Yarizadeh, H.; Setayesh, L.; Majidi, N.; Rasaei, N.; Mehranfar, S.; Ebrahimi, R.; Casazzza, K.; Mirzaei, K.	2022	Nutrient patterns and their relation to obesity and metabolic syndrome in Iranian overweight and obese adult women
482	Yoo, K. B.; Suh, H. J.; Lee, M.; Kim, J. H.; Kwon, J. A.; Park, E. C.	2014	Breakfast eating patterns and the metabolic syndrome: the Korea National Health and Nutrition Examination Survey (KNHANES) 2007-2009
483	Yoo, S.; Nicklas, T.; Baranowski, T.; Zakeri, I. F.; Yang, S. J.; Srinivasan, S. R.; Berenson, G. S.	2004	Comparison of dietary intakes associated with metabolic syndrome risk factors in young adults: the Bogalusa Heart Study
484	Young, J. M.; Florkowski, C. M.; Molyneux, S. L.; McEwan, R. G.; Frampton, C. M.; Nicholls, M. G.; Scott, R. S.; George, P. M.	2012	A randomized, double-blind, placebo-controlled crossover study of coenzyme Q10 therapy in hypertensive patients with the metabolic syndrome
485	Yu, S.; Wang, B.; Li, G.; Guo, X.; Yang, H.; Sun, Y.	2023	Habitual Tea Consumption Increases the Incidence of Metabolic Syndrome in Middle-Aged and Older Individuals

486	Yubero-Serrano, E. M.; Delgado-Lista, J.; Tierney, A. C.; Perez-Martinez, P.; Garcia-Rios, A.; Alcala-Diaz, J. F.; Castaño, J. P.; Tinahones, F. J.; Drevon, C. A.; Defoort, C.; Blaak, E. E.; Dembinska-Kieć, A.; Risérus, U.; Lovegrove, J. A.; Perez-Jimenez, F.; Roche, H. M.; Lopez-Miranda, J.	2015	Insulin resistance determines a differential response to changes in dietary fat modification on metabolic syndrome risk factors: the LIPGENE study
487	Zamanillo-Campos, R.; Chaplin, A.; Romaguera, D.; Abete, I.; Salas-Salvadó, J.; Martín, V.; Estruch, R.; Vidal, J.; Ruiz-Canela, M.; Babio, N.; Fiol, F.; de Paz, J. A.; Casas, R.; Olbeyra, R.; Martínez-González, M. A.; García-Gavilán, J. F.; Goday, A.; Fernandez-Lazaro, C. I.; Martínez, J. A.; Hu, F. B.; Konieczna, J.	2022	Longitudinal association of dietary carbohydrate quality with visceral fat deposition and other adiposity indicators
488	Zandvakili, A.; Shiraseb, F.; Hosseininasab, D.; Aali, Y.; Santos, R. D.; Mirzaei, K.	2024	The association between consumption of red and processed meats with metabolic syndrome and its components in obese and overweight women: a cross-sectional study
489	Zaribaf, F.; Falahi, E.; Barak, F.; Heidari, M.; Keshteli, A. H.; Yazdannik, A.; Esmaillzadeh, A.	2014	Fish consumption is inversely associated with the metabolic syndrome
490	Zilaee, M.; Kermany, T.; Tavalaee, S.; Salehi, M.; Ghayour-Mobarhan, M.; Ferns, G. A.	2014	Barberry treatment reduces serum anti-heat shock protein 27 and 60 antibody titres and high-sensitivity c-reactive protein in patients with metabolic syndrome: a double-blind, randomized placebo-controlled trial

WDI Supplementary Figures:

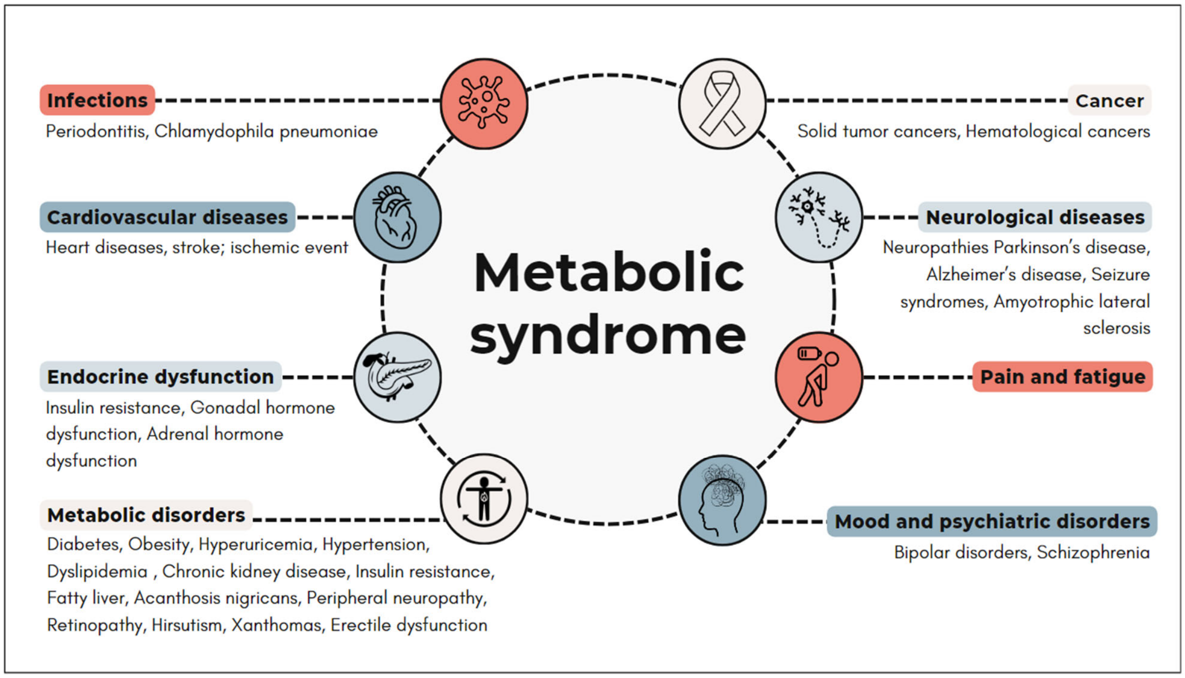

Supplementary Figure S1. Metabolic syndrome comorbidities, adapted from Integrative and functional medical nutrition therapy book, chapter 2, pages 17-29 [2], showing MetS as a risk factor for various health-related conditions including CVDs and T2D, among others.

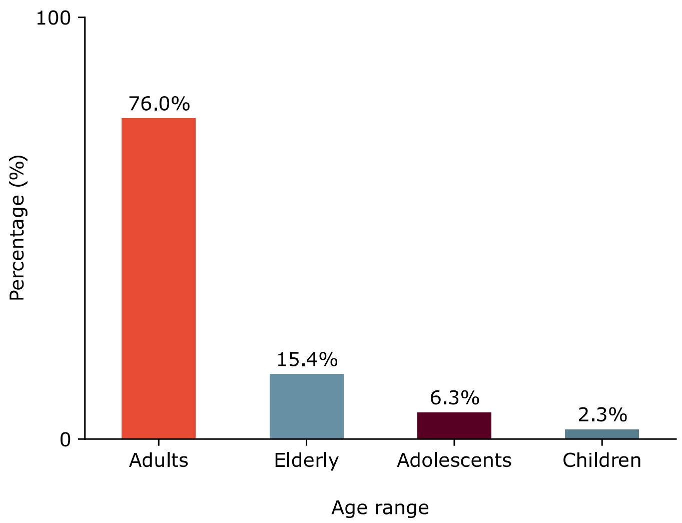

Supplementary Figure S2. Bar chart of age distribution in the selected studies to develop the WDI. Adults: 18-65 years, Elderly: >65 years, Adolescent: 10-18 years, Children: (18 months to 10 years)

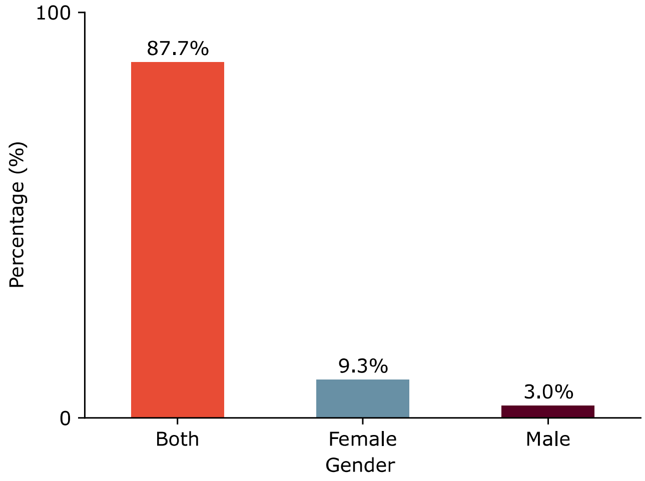

Supplementary Figure S3. Bar chart of gender distribution in the selected studies to develop the WDI

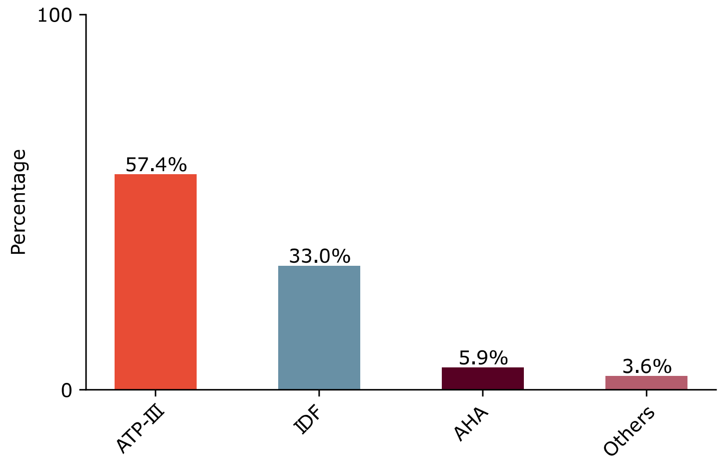

Supplementary Figure S4. Bar chart of MetS definition distribution in the selected studies to develop the WDI
